# Deciphering the tumor immune microenvironment: single-cell and spatial transcriptomic insights into cervical cancer fibroblasts

**DOI:** 10.1186/s13046-025-03432-5

**Published:** 2025-07-05

**Authors:** Zhiheng Lin, Youwei Zhou, Zhenran Liu, Wenyang Nie, Hengjie Cao, Shengnan Li, Xuanling Li, Lijun Zhu, Guangyao Lin, Yanyu Ding, Yi Jiang, Zuxi Gu, Lianwei Xu, Zhijie Zhao, Huabao Cai

**Affiliations:** 1 Department of Gynecology, Longhua Hospital, Shanghai University of Traditional Chinese Medicine, Shanghai, 200032 China; 2 Department of Obstetrics and Gynecology, the First Affiliated Hospital of Anhui Medical University, Hefei, 230032 China; 3 The First Clinical Medical College, Shandong University of Traditional Chinese Medicine, Jinan, 250014 China; 4 Department of Immunology, School of Basic Medical Sciences, Center for Big Dataand , Population Health of IHM, Anhui Medical University, Hefei, 230032 China; 5 Institute of Health and Medicine, Hefei Comprehensive National Science Center, Hefei Economic and Technological Development Zone, 4090 Susong Rd, HefeiHefei, Anhui 230601 China; 6 Experiment Center for Science and Technology, Shanghai University of Traditional Chinese Medicine, Shanghai, 201203 China; 7 Department of Laboratory Animal, School of Experimental Center of Science and Technology, Shanghai University of Traditional Chinese Medicine, Shanghai, 201203 China; 8 Department of Plastic and Reconstructive Surgery, School of Medicine, Shanghai Ninth People’ S Hospital, Shanghai Jiao Tong University, Shanghai, China; 9 Department of Neurosurgery, The First Affiliated Hospital of Anhui Medical University, 218 Jixi Rd, Hefei Shushan Zone, Hefei, Anhui 230032 China; 10 Center for Scientific Researchof, Anhui Medical University, Anhui Medical University, Hefei Shushan Zone, 81Meishan Rd, Hefei, Anhui 230032 China

**Keywords:** Cervical cancer, Tumor immune microenvironment, Single-cell RNA sequencing, Spatial transcriptomics, Cancer-associated fibroblasts, SDC1, Immunomodulation

## Abstract

**Background:**

Cervical cancer (CC) remains a significant global health challenge despite advancements in screening, HPV vaccination, and therapeutic strategies. Tumor heterogeneity, driven by epigenetic modifications, affects immune evasion, metastasis, and treatment response. Cancer-associated fibroblasts (CAFs) play a crucial role in CC progression and therapy resistance. Single-cell sequencing offers new insights but remains underutilized in CC research. This study integrates single-cell RNA sequencing (scRNA-seq), spatial transcriptomics, and deconvolution analysis to identify key genes and immunotherapy targets. By constructing a prognostic model and exploring the immune microenvironment, we aim to provide novel insights into CC pathogenesis and potential therapeutic strategies.

**Methods:**

We utilized scRNA-seq, spatial transcriptomics, deconvolution analysis, and pseudotime trajectory mapping to delineate fibroblast subtypes within the tumor immune microenvironment (TIME) of CC. Functional annotations, differential gene expression profiling, cell–cell communication pathways, and transcription factor networks were systematically analyzed. A prognostic model based on bulk RNA-seq data was constructed and validated through survival analysis, with correlations to immune microenvironment characteristics. Functional experiments investigated the role of SDC1, a critical mediator of fibroblast-tumor crosstalk. Additionally, Fibroblast–tumor cell co-culture systems and functional assays were employed to investigate the paracrine role of SDC1. The CAF MYH11⁺ subpopulation was isolated via fluorescence-activated cell sorting (FACS). Multiplex immunofluorescence and immunohistochemical analyses were performed on both cultured cells and human cervical cancer tissue samples to characterize the spatial distribution and dynamic remodeling of MYH11 during stromal reorganization.

**Results:**

Six distinct fibroblast subtypes were identified, including the C0 MYH11 + fibroblasts, which exhibited unique roles in stemness maintenance, metabolic activity, and immune regulation. Spatial and functional analyses revealed that the C0 subtype is central to tumor-fibroblast interactions, particularly through the MDK-SDC1 signaling axis. The prognostic model incorporating fibroblast-specific markers demonstrated robust predictive power for patient survival outcomes. Additionally, in vitro SDC1 knockdown significantly inhibited CC cell proliferation, migration, and invasion. Fibroblasts show spatially regulated heterogeneity, with activation markers enriched in the tumor zone and MYH11 highest in normal zones, indicating dynamic stromal remodeling. C0 MYH11 + CAF Promotes Tumor Cell Proliferation, Migration, and Inhibits Apoptosis via Soluble SDC1.

**Conclusion:**

Our results illustrate, in some ways, the possible immunomodulatory and tumor supporting roles of CAFs in CC TIME and highlight the possibility that the MDK-SDC1 pathway is a promising therapeutic target. This study not only promotes a partially new understanding of temporal heterogeneity in CC, but also provides a possible reference base for the development of new biomarkers and immunotherapy approaches to improve clinical outcomes.

**Supplementary Information:**

The online version contains supplementary material available at 10.1186/s13046-025-03432-5.

## Introduction

Cervical cancer (CC) ranks as the fourth most commonly diagnosed cancer and the fourth leading cause of cancer-related death in women worldwide. In transitioning countries, its incidence and mortality rates are second only to breast cancer [1]. The predominant histological subtypes of CC include squamous cell carcinoma (SCC), adenocarcinoma (AC), and adenosquamous carcinoma (ASC). Despite advancements in CC screening, widespread adoption of HPV vaccination, and improvements in diagnostic and therapeutic strategies, CC continues to pose a significant public health challenge [2]. Understanding the molecular mechanisms underlying cervical carcinogenesis is therefore crucial for developing novel therapeutic approaches and improving patient survival outcomes.

The heterogeneity of CC underpins effective prevention and precise treatment. Tumor heterogeneity manifests across multiple dimensions, including immune evasion, recurrence, metastasis, and drug resistance [3]. Epigenetic modifications during tumor development and progression influence tumor cell proliferation, invasion, and therapy response, thereby shaping the heterogeneity of the tumor microenvironment (TME) [4, 5]. This heterogeneity directly impacts diagnostic accuracy, treatment efficacy, and prognosis [6, 7]. Hence, a comprehensive understanding of TME heterogeneity is essential to improving CC prognosis. Currently, international research on cervical cancer has made important breakthroughs in the mechanism of occurrence, immunotherapy, novel therapeutic targets and multi-omics integration. It has been found that the integration of HPV DNA into the host genome can cause DNA breaks, gene amplification, and activation of oncogene expression, such as MYC and TERT [8, 9]. It also regulates host gene expression through DNA methylation and histone modification, thus affecting the process of carcinogenesis [10]. High expression of PD-L1 and upregulation of T-cell depletion markers (e.g. TIM-3, LAG-3) in cervical cancer tissues suggests the possibility of combination immunotherapy [11]. Proteomic and transcriptomic analyses showed that aberrant activation of NOTCH1 and WNT pathways may be associated with increased invasiveness of cervical cancer cells [12]. Genomic and metabolomic analyses showed that the glycolytic pathway was highly activated in cervical cancer patients, suggesting that metabolic intervention may be a new therapeutic strategy [13].

The advent of single-cell sequencing technology has provided a powerful tool to unravel TME heterogeneity, offering new avenues for therapeutic innovation [14–16]. However, the application of single-cell sequencing in oncology remains limited to a few tumor types. Research on CC using single-cell sequencing is sparse, resulting in a limited understanding of its intratumoral heterogeneity.

Cancer-associated fibroblasts (CAFs), as critical components of the CC TME, play pivotal roles in tumor progression, treatment resistance, and immune evasion. For instance, Takuya et al. recreated a metastatic TME by co-transplanting human CC cells with CAFs into athymic mice, leading to lymph node metastases in 40% of the cases, whereas CC cells alone failed to metastasize [17]. These findings highlight the role of CAFs in overcoming anti-metastatic barriers and underscore their potential as therapeutic targets. Similarly, Liu et al. identified key CAF-associated genes—COL4 A1, LAMC1, RAMP3, POSTN, and SERPINF1—through weighted gene co-expression network analysis (WGCNA) and developed a predictive model based on these markers [18]. Such studies emphasize the significance of CAFs in CC development and their potential as therapeutic targets.

In this study, we aimed to mine key genes and immunotherapy targets that may be associated with CC initiation and progression by integrating single-cell RNA sequencing (scRNA-seq), deconvolution, spatial transcriptomics, and pseudotemporal analysis. In addition, we will construct a prognostic model based on a large number of RNA-seq data, explore the CC immune microenvironment, and explore the possible reference value of many previous reference data through a series of experiments. Our goal is to provide new possible insights and references for immunotherapy strategies to promote CC and hopefully to be able to ultimately contribute to improving patient outcomes.

## Methods

### Data collection and processing

**Fig. 1:**
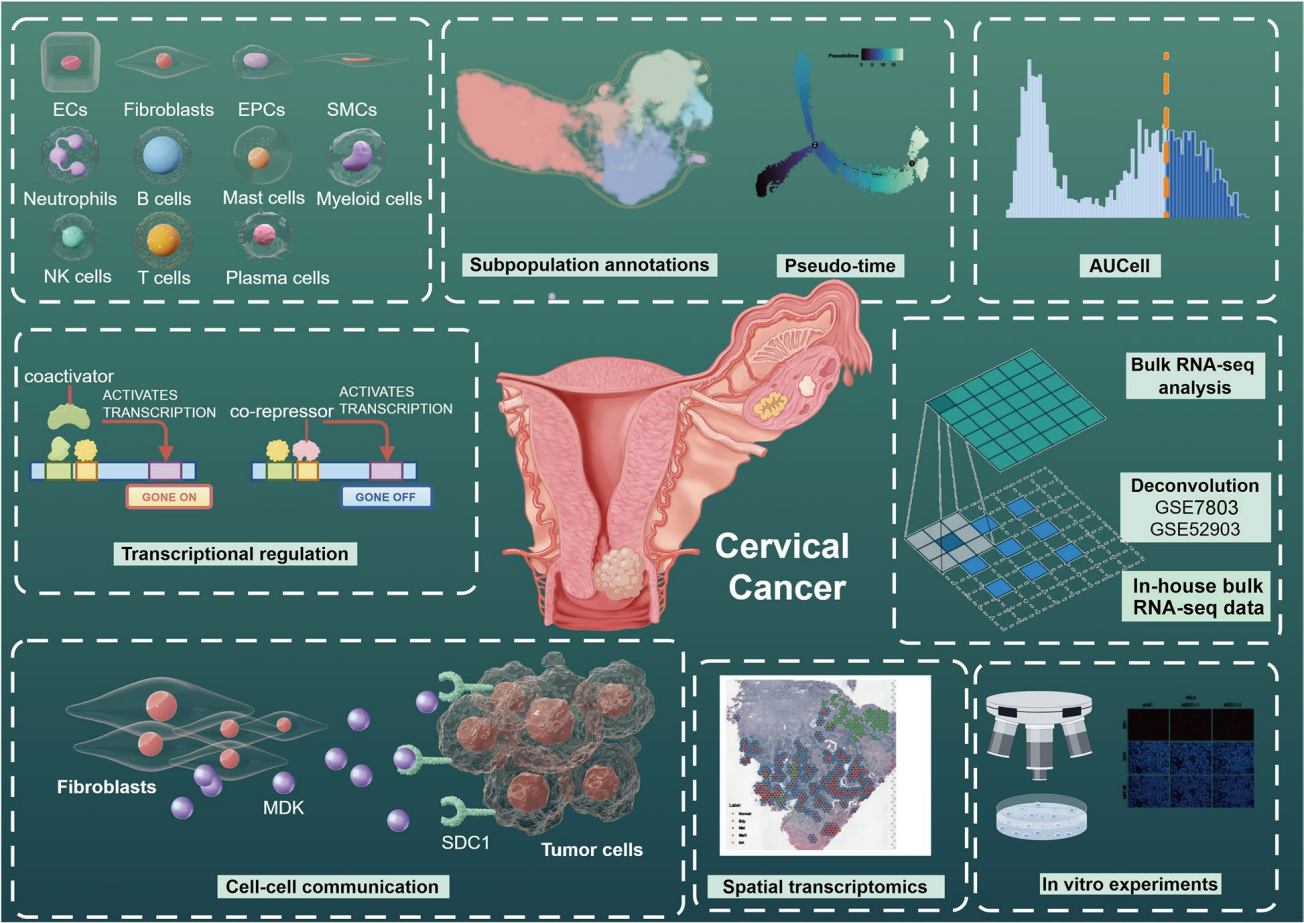
The overview of this study

The comprehensive workflow and methodology of this study are summarized in Fig. 1. The scRNA-seq data were obtained from the https://www.ebi.ac.uk/biostudies/arrayexpress under the accession number E-MTAB-12305 and the bulk data were obtained from The Cancer Genome Atlas (TCGA) website (https://portal.gdc.cancer.gov/) and UCSC Xena (https://xena.ucsc.edu/) and the Gene Expression Omnibus (GEO) (https://www.ncbi.nlm.nih.gov/geo/). The spatial transcriptome data from 10 × website (https://www.10xgenomics.com/).

The 10 × genomic data for each sample were loaded into the R software (v4.3.0) using the Seurat package (v4.3.0). Initially, potential duplicates and cells of low quality were filtered using the DoubletFinder program (v2.0.3). Cells were retained for further analysis if they met the following criteria: 500 < nCount < 50000,300 < nFeature < 7000 and mitochondrial gene expression was below 25% of the total count.

### Identifying the cell type

After normalizing the selected samples, we identified the top 2000 high-variance genes (HVGs) from the resulting expression matrix. The ScaleData function standardized the data. These HVGs were analyzed through principal component analysis (PCA), with the first 30 principal components (PCs) selected for further processing. Batch effects between samples were corrected using the Harmony method (v1.2.0). FindClusters and FindNeighbors functions are used to identify clusters. The data were then visualized in two dimensions via UMAP to facilitate cell type identification. Cell markers were obtained from the CellMarker database (http://xteam.xbio.top/CellMarker/) and applied to annotate the cell clusters, allowing us to define distinct cell types and evaluate their distribution and proportions. Additionally, to uncover the heterogeneity among CC fibroblasts, we performed reclustering and annotated each fibroblast subpopulation based on its specific gene expression patterns.

### Enrichment analysis of differential gene expression and AUCell

To identify differentially expressed genes (DEGs) in each cell type, we utilized the ‘FindAllMarkers’ function, employing the Wilcoxon rank sum test with default parameters (Log FC > 0.25). Functional roles of the DEGs were further explored through enrichment analyses performed using the clusterProfiler (v4.6.2) and SCP (v0.4.8) packages. Pathway analysis for each cell type was conducted using GO-BP and GSEA. To evaluate pathway activity at the single-cell level, active genes within single-cell RNA-seq data were detected using the AUCell (v1.22.0) tool.

### Determination of cellular CNV levels

We calculated CNV levels using the InferCNV (v1.16.0) algorithm. Copy number karyotyping of aneuploid cells during tumorigenesis was used to differentiate between non-tumor cells and malignant tumor cells. Endothelial cells were used as an inferCNV reference to determine whether other cancer cells exhibited substantial chromosomal copy number variation. We calculated the CNV status of the fibroblasts. Furthermore, we further screened out tumor cells from EPCs.

### Pseudotime trajectory of CC fibroblasts subtypes

Pseudotemporal trajectory analysis of CC cells was conducted using the Monocle package (v2.24.0). Based on pseudotemporal profiles derived from scRNA-seq data, Monocle facilitated the identification of cellular transitions linked to CC fibroblast differentiation.

### Slingshot

To explore the differences in the developmental and differentiation status of different subpopulations of CC fibroblasts, we first constructed trajectories reflecting the developmental stages and status of each subpopulation using the ‘ Slingshot’ software package (v2.8.0).

### Cell–cell communication and pySCENIC analysis

Potential interactions between cell types were predicted using single-cell RNA sequencing data and the CellChat software package (v1.6.1). CellChatDB.human is the reference database for ligand-receptor interactions. We used a significance threshold with a P-value cut-off of 0.05 in this database to predict cell–cell interactions between different cell types. PySCENIC is a tool for reconstructing gene regulatory networks (GRN) based on scRNA-seq data, with a special focus on identifying stable cellular states. We applied the pySCENIC (v0.12.1) tool in Python (v3.9.0) using default parameters to perform regulatory network reconstruction and evaluate transcription factor activities. AUCell matrices were generated to assess the enrichment of transcription factors and the activity of their corresponding regulators, providing insight into the regulatory environment that controls cell state and transition.

### Constructing the prognostic model for CC fibroblasts

Transcriptomic data of CC patients with comprehensive clinical information were retrieved from the TCGA database (https://portal.gdc.cancer.gov/) and UCSC Xena (https://xena.ucsc.edu/) database for further analysis. In order to investigate the potential of CC fibroblasts as predictors of patient survival, we employed a strategy that involved utilising marker genes for key subtypes of fibroblasts as predictor genes. Univariate Cox and Lasso regression analyses were conducted using the 'survival' R package to identify additional key prognostic genes. Prognostic models were subsequently developed through multivariate Cox regression analyses. Risk scores for each sample were determined using the formula:

$$\mathrm{Riskscore}=\:(\mathrm{ExpressionGene}1\times\mathrm{Coefficient}1)+(\mathrm{ExpressionGene}2\times\mathrm{Coefficient}2)+\dots+(\mathrm{ExpressionGeneN}\times\mathrm{CoefficientN}).$$

Based on the optimal cut-off value of the MYH11+ fibroblast risk score (MFRS), the CC cohort was divided into two groups: a high MFRS group and a low MFRS group. 

### Bulk transcriptome analysis

To assess differences in survival, we plotted Kaplan–Meier curves for survival analysis using the survival software package (v3.5–5) and the survminer package (v0.4.9). To assess and calibrate the predictive accuracy of the model, receiver operating characteristic (ROC) curves were generated for 1-, 3-, and 5-year intervals using the'timeROC'software package (v0.4). PCA analysis was performed to visualize the transcriptomic differences between the high and low MFRS groups. To evaluate the prognostic value of the MFRS, multivariate Cox regression analysis was performed incorporating clinical variables. A nomogram was constructed using the rms (v6.7-1) package to assess the predictive power of the risk score. Pearson correlation analysis was used to explore associations between key molecular features. In addition, immune-related analyses were performed to characterize the tumor immune microenvironment. Somatic mutation and copy number variation (CNV) analyses were conducted to compare genomic alterations between the two groups. And drug sensitivity analysis was conducted using the pRRophetic (v0.5) package to predict potential therapeutic responses between groups. Differential expression analysis between the high and low MFRS groups was performed using the DESeq2 (v1.42.1) package. Enrichment analysis of differentially expressed genes (DEGs) was conducted using clusterProfiler for Gene Ontology (GO) and Kyoto Encyclopedia of Genes and Genomes (KEGG) pathways. To further explore biological relevance, Gene Set Enrichment Analysis (GSEA) was carried out. In addition, GSVA (v1.50.5) was used to score key pathways and assess their activity across samples. 

### Spatial transcriptomics sequencing analysis

We obtained the CC spatial transcriptomics dataset from the 10 × Genomics website (https://www.10xgenomics.com/). Initial data processing was performed using the Seurat package. The dataset was normalized and log-transformed using the ‘SCTransform’ function. Dimensionality reduction was conducted with the ‘RunPCA’ function. To map the spatial distribution of cell populations, the integrated scRNA-seq dataset was used as a reference. Cell type deconvolution was conducted using the spacexr (v2.2.1) package with the Robust Cell Type Decomposition (RCTD) method, enabling estimation of cell type proportions at each spatial spot. To further characterize the tumor microenvironment and delineate tumor boundaries within the spatial transcriptomics slices, we utilized the Cottrazm (v0.1.1) package. This allowed for the spatial annotation of tumor versus non-tumor regions based on transcriptional patterns and cell type composition. In addition, key genes and pathways were further examined by visualizing their spatial expression patterns, highlighting region-specific biological activity across the tissue. Furthermore, spatial interactions between tissue regions were analyzed using the stlearn (v0.4.12) tool to investigate spatial communication and microenvironmental organization.

### Deconvolution

The Bulk data used for deconvolution comes from the GEO database. The GSE accession is GSE7803 and GSE52903. In addition, we also included in-house bulk RNA-seq transcriptome data (three tumor groups and three paracancerous control groups). To understand the pathological process of CC, we deconvolved bulk RNA-seq samples at the level of a wide range of cell types, using scRNA-seq data as a reference. A full set of common genes and selected marker genes were entered into the BayesPrism package (v2.2.2) to deconvolute the data.

### GeneSwitches analysis

To elucidate the sequence of key gene expression and functional events during CC fibroblast activation, we used the GeneSwitches (v0.1.0) computational tool in conjunction with pseudo-temporal trajectory analysis of scRNA-seq data. GeneSwitches identifies ‘switch genes’ that these genes act as on/off regulators between cell states and determine the sequence of these transitions. GeneSwitches performs pathway analyses using the KEGG and GO datasets, which are collections of signature pathways and genomes in the Molecular Signatures Database (MSigDB). GeneSwitches also supports comparative analysis of two related pseudo-temporal trajectories by identifying and visualizing common switching genes. In addition, it highlights switch genes unique to each track, providing insight into pathway or genome specificity.

### CytoTRACE and CytoTRACE 2 analysis

We analyzed scRNA-seq data to predict CC fibroblast potential categories and absolute developmental potential using CytoTRACE (v0.3.3) and CytoTRACE2 (v1.0.0). CytoTRACE ranks the differentiation status of all fibroblast subsets. The potential categories in CytoTRACE2 enable classification of cells according to their developmental potential, and in addition, the predicted potential values provide continuous measures of developmental potential ranging from 0 (differentiation) to 1 (totipotency). Facilitates direct cross-dataset comparisons of developmental potential in absolute space.

### Cell culture

The CC cell lines HeLa and SiHa were sourced from Procell (Wuhan, China). The cells were cultured in MEM medium supplemented with 10% fetal bovine serum (FBS), 100 U/mL penicillin, and 0.1 mg/mL streptomycin. Cultures were maintained in a humidified incubator at 37 °C with 5% CO₂. Cells in the logarithmic growth phase were harvested for subsequent experiments. Additionally, HT-3 and ME-180 cervical cancer cell lines were obtained from the Cell Bank of the Chinese Academy of Sciences (Shanghai, China). HT-3 cells were cultured in McCoy’s 5A medium (Gibco, USA) supplemented with 10% FBS, 100 U/mL penicillin, and 0.1 mg/mL streptomycin. ME-180 cells were maintained in Eagle’s Minimum Essential Medium (EMEM; Gibco, USA) with 10% FBS and antibiotics under identical incubation conditions. All cell lines were routinely tested for mycoplasma contamination.

### Human tissue specimen collection

Human cervical cancer and adjacent normal tissue samples were obtained from patients undergoing gynecological surgery at the Department of Obstetrics and Gynecology, The First Affiliated Hospital of Anhui Medical University (Hefei, China). All patients provided written informed consent prior to sample collection. The study protocol was reviewed and approved by the Ethics Committee of The First Affiliated Hospital of Anhui Medical University (Approval No.PJ2021-15-28). Fresh surgical specimens were immediately processed for primary cell isolation, histological analysis, or cryopreservation according to experimental requirements. All procedures involving human tissues were conducted in accordance with the principles of the Declaration of Helsinki and institutional ethical standards.

### SiRNA transfection

For the experimental procedure, cells were seeded onto 6-well plates at a density of 2 × 10^5 cells per well. After 24 h, the cells were transfected with siRNAs obtained from GenePharma (Shanghai, China) at a final concentration of 20 µM. Transfection was performed using RNAiMax (Life Technologies, ThermoFisher Distributor;Brendale QLD,Australia) following the manufacturer's instructions. Cells were collected 24 h post-transfection for further analysis. The sequences of the SDC1 siRNAs used in the experiment were as follows:

$$\mathrm{siRNA}1:\mathrm{GACUGCUUUGGACCUAAAU};\mathrm{siRNA}2:\mathrm{GCAAAUUGUGGCUACUAAU}.$$

### RNA extraction and Quantitative real-time PCR

Total RNA was extracted using TRIzol reagent (Thermo Fisher Scientific, Waltham, MA, USA) according to the manufacturer's protocol. Next, 500 ng of RNA was reverse-transcribed into cDNA with the PrimeScript RT Reagent Kit (TaKaRa, Tokyo, Japan). Quantitative real-time PCR was performed using the SYBR® Premix Ex Taq™ (TaKaRa). PCR amplification was carried out on the ABI V7 instrument (ABI, Indianapolis, IN, USA). The specific primers used for amplification were designed for the SDC1 gene as follows:Forward primer:5’-CCAAGCTGACCTTCACACTC-3’ and Reverse primer:5’-GGCCACTACAGCCGTATTCT-3’.

### Cell viability assay

Cell viability was evaluated using the Cell Counting Kit-8 (CCK-8) from DOJINDO (Kumamoto, Japan). Cells were seeded at 1 × 10^3 cells per well in 96-well plates and cultured overnight. A 100 µL detection reagent was added to each well and incubated for 1 h. Absorbance at 450 nm was measured daily over 4 days. Growth curves were plotted by correlating OD450 values with time to assess cell viability.

### Clone formation assay

Suspensions of logarithmically growing cells were prepared and diluted, with 1 × 10^3 cells plated per well in 6-well plates. Cells were cultured for 10 days, with regular observations. Once colonies became visible, the culture medium was removed, and cells were washed twice with ice-cold PBS before being fixed in 4% paraformaldehyde for 20 min. After fixation, cells were stained with 0.1% crystal violet for 10 min, and colonies were counted using the Gel Imaging Analysis System (Syngene, GBOX-F3EE; Bangalore, India).

### EdU analysis

Logarithmically growing cell suspensions were prepared, diluted, and seeded at a density of 1 × 10^3 cells per well in 6-well plates. The EdU assay was carried out following the manufacturer's instructions (RiboBio, China). Treated cells were examined under a fluorescence microscope, and the number of EdU-positive cells was quantified by counting cells in a minimum of six randomly selected fields.

### Wound healing assay

2 × 10^5 cells per well were seeded in 6-well plates and cultured overnight. A scratch was made in the cells with a 10 µL pipette tip held perpendicular to the culture plate. Cells were rinsed with PBS three times to remove detached cells, and fresh serum-free medium was added back to the plates. Cells were incubated at 37 °C in a chamber with 5% CO2, and images were acquired at 0 h and 48 h under bright field microscopy.

### Transwell migration and invasion assay

Transwell assays were conducted to evaluate cell migration and invasion. Cells were seeded in 24-well Transwell insert chambers (BD Biosciences, USA) with serum-free medium. For the invasion assay, the inserts were pre-coated with 2% Matrigel. FBS (20%) was added to the lower chamber as a chemoattractant. After 48 h, cells that did not migrate, located on the upper surface, were removed. Migrated cells on the lower surface were then fixed, stained with crystal violet, and examined under a microscope for imaging and analysis.

### MYH11 + fibroblasts isolation

Primary CAFs were isolated from fresh human tumor specimens using a Tumor Dissociation Kit (Miltenyi Biotec, Cat# 130–095–929), which includes optimized enzymatic reagents (collagenase IV, hyaluronidase, and DNase I) for efficient tissue digestion. Tumor tissues were minced into ~ 1–3 mm^3^ fragments and enzymatically dissociated at 37 °C for 45 min with gentle agitation, followed by filtration through a 70 μm cell strainer to obtain single-cell suspensions. CAFs were initially enriched by differential adhesion (6–12 h) and further purified using anti-fibroblast magnetic beads (STEMCELL Technologies, Cat# 17,859). To eliminate contaminating cells, epithelial (EpCAM +), endothelial (CD31 +), and hematopoietic (CD45 +) cells were depleted using magnetic beads (Miltenyi Biotec, Cat# 130–105–958, 130–091–935, and 130–045–801, respectively). For isolation of MYH11 + CAF subpopulations, cells were permeabilized using BD Cytofix/Cytoperm™ (BD Biosciences, Cat# 554,714), followed by intracellular staining with anti-MYH11-Alexa Fluor 647 (Abcam, Cat# ab53219) and surface staining with anti-α-SMA-PE (R&D Systems, Cat# IC1420P), anti-CD31-APC, anti-CD45-FITC, and anti-EpCAM-BV421 (BioLegend, Cat# 303,115, 304,005, and 324,215). Flow cytometry was performed on a BD FACSAria III (BD Biosciences), and MYH11 + CAFs were sorted using a sequential gating strategy: live single cells → α-SMA + → MYH11 +. Purity was confirmed by re-analysis of sorted populations.

### mIF

Multicolor immunofluorescence staining was performed to detect α-SMA, Vimentin, Calponin, Fibronectin, and MYH11. Tissue sections or cultured cells were fixed with 4% paraformaldehyde (Sigma-Aldrich, USA) for 15 min at room temperature (RT). When necessary, antigen retrieval was carried out using citrate buffer (pH 6.0) at 95 °C for 15 min. After fixation, samples were permeabilized with 0.3% Triton X-100 (Sigma-Aldrich, USA) for 10 min, followed by blocking with 5% bovine serum albumin (BSA, Sigma-Aldrich, USA) for 1 h at RT to reduce non-specific binding. The samples were then incubated overnight at 4 °C with primary antibodies purchased from Abcam (Cambridge, UK): α-SMA (ab32575, 1:200), Vimentin (ab92547, 1:200), Calponin (ab46794, 1:200), Fibronectin (ab23750, 1:200), and MYH11 (ab133567, 1:200). After washing with PBS three times, Alexa Fluor-conjugated secondary antibodies (Alexa Fluor 488-conjugated goat anti-rabbit IgG [ab150077, 1:500] and Alexa Fluor 594-conjugated goat anti-mouse IgG [ab150116, 1:500], Abcam) were applied for 1 h at RT in the dark. Nuclei were counterstained with DAPI (ab228549, 1:1000, Abcam) for 10 min before mounting with Fluoromount-G (SouthernBiotech, USA). Imaging was performed using a Zeiss LSM 880 confocal microscope (Germany), and co-localization analysis was conducted using Pearson’s correlation coefficient with ImageJ software (NIH, USA).

### mIHC

Formalin-fixed, paraffin-embedded (FFPE) human tumor tissues, adjacent normal tissues, and tumor border zone tissues were sectioned at 4 μm. Slides were deparaffinized with xylene, rehydrated through graded ethanol, and subjected to heat-induced antigen retrieval using 10 mM citrate buffer (pH 6.0) in a pressure cooker. For multiplex immunohistochemistry (mIHC), sequential single-antibody staining with tyramide signal amplification (TSA) was performed. Endogenous peroxidase activity was blocked with 3% H₂O₂, followed by blocking with 5% normal serum. Primary antibodies, including anti-α-SMA (Abcam, Cat# ab5694), anti-Vimentin (Cell Signaling Technology, Cat# 5741), anti-Calponin (Abcam, Cat# ab46794), anti-Fibronectin (Abcam, Cat# ab2413), and anti-MYH11 (Abcam, Cat# ab53219), were applied sequentially. After incubation, slides were treated with HRP-conjugated secondary antibodies (Jackson ImmunoResearch, species-specific), followed by TSA fluorophore labeling using individual fluorophores (PerkinElmer, Cat# FP1487001 KT, FP1497001 KT). Between each round, antibody stripping was performed using 0.1 M glycine buffer (pH 2.0) at 50 °C. After the final staining cycle, nuclei were counterstained with DAPI (Thermo Fisher Scientific, Cat# D1306), and slides were mounted using ProLong™ Gold Antifade Mountant (Thermo Fisher Scientific, Cat# P36930). Imaging was conducted on a Vectra® Polaris™ Automated Quantitative Pathology Imaging System, and spectral unmixing and quantification were performed using inForm® software (Akoya Biosciences).

### Cell transfection

Transfections were performed using the Lipofectamine 3000 Kit (Invitrogen Carlsbad, CA, USA) according to the manufacturer’s instructions. Cells were grown to 50% to 60% confluence in 6-well plates and transfected with plasmid containing shRNAs. After infection for 72 h, cells were selected in the presence of 2 μg/mL puromycin, and cells resistant to puromycin were collected and cultured. The construction of stable cell lines was completed. Cells were harvested 72 h after transfection for subsequent confirming the transfection efficiency by quantitative real-time polymerase chain reaction (qPCR) or Western blotting analysis.

### RNA Isolation and qPCR

Total RNA was obtained from tissues or cells by using the TRIzol reagent (Invitrogen Corporation, Waltham, MA, USA) according to the manufacturer’s instructions. The concentration and purity of the RNA were assessed by using a NanoDrop 2000 instrument (Thermo Scientific, Waltham, MA, USA). First-strand cDNA was synthesized from total RNA by using a GoScript Reverse Transcription System (Promega, Madison, WI, USA). qPCR was performed using GoTaq qPCR Master Mix Kit (Promega, Madison, WI, USA). GAPDH was used as an endogenous control for normalization. All primers used in this study were customized by Genscript. The primer sequences are presented in Supplementary Table 1.

### Sphere formation assay

Tumor cells were treated with control medium, co-culture with CAF^MYH11+^ or supernatant collected from CAF^MYH11+^ treated with indicated conditions for 24 h. After 72-h incubation, a total of 1000 single cells per well were seeded in ultra-low attachment 6-well plates in DMEM/F12 medium (Sigma-Aldrich) containing B27 supplement (Gibco, Invitrogen Carlsbad, CA, USA), EGF (20 ng/mL; Peprotech) and bFGF (20 ng/mL; Peprotech). After 7 days, spheres with a diameter > 75 μm were counted.

### Western blotting

Total cell proteins were extracted using radioimmunoprecipitation assay (RIPA) lysis buffer. The protein concentration was measured using the bicinchoninic acid (BCA) assay method (Biyuntian, Jiangsu, China). Equal amounts of proteins were separated on a 10% SDS–polyacrylamide gel electrophoresis (SDS-PAGE) gel. After electrophoresis, proteins were transferred onto a polyvinylidene fluoride (PVDF) membrane. The membrane was blocked with 5% nonfat milk for 1 h at room temperature (RT), followed by incubation with primary antibodies overnight at 4 °C. Afterward, the membrane was incubated with the appropriate secondary antibody for 2 h at 37 °C. The following primary antibodies from Abcam (Cambridge, UK) were used: SDC1 (ab142138, 1:1000), β-actin (ab8227, 1:5000).The secondary antibody used was horseradish peroxidase (HRP)-conjugated anti-rabbit IgG (ab6721, 1:5000, Abcam). The protein bands were detected using the enhanced chemiluminescence (ECL) detection system (Thermo Fisher, USA), and image analysis was performed using the ImageJ software (NIH, USA).

### Statistical analysis

Statistical analysis was performed using R software (v4.3.0) and Python software (v3.9.0). Wilcoxon’s test and Pearson’s correlation coefficient were used to assess the significance of differences between groups. Significance levels were defined as follows: *P < 0.05, **P < 0.01, ***P < 0.001, ****P < 0.0001, with 'ns' indicating non-significant differences. These statistical tests and significance thresholds were applied to evaluate the reliability of the findings and ensure robust results.

## Results

### CC Fibroblast Subtype Landscape

**Fig. 2:**
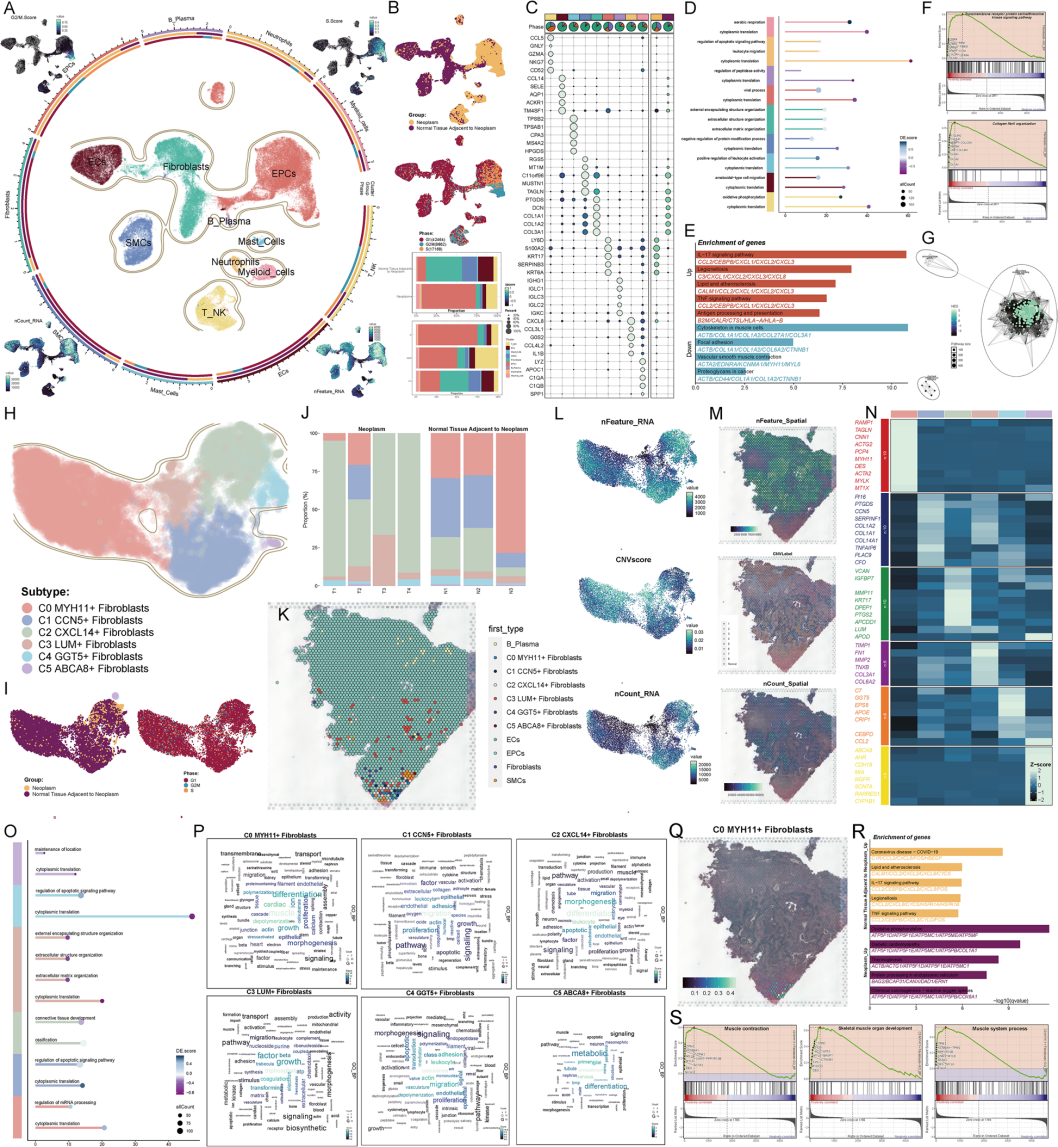
Characterization of fibroblast subtypes in cervical cancer. **A** All high-quality cells were dimensionally reduced and clustered into UMAP plots, with cell types annotated based on differentially expressed genes. The differences in G2/M score, S score, nCount RNA, and nFeature RNA across cells were also displayed. **B** UMAP plots showed the distribution of cells from the Neoplasm group and the Normal tissue adjacent to neoplasm group. The cell cycle phase distributions within all annotated cell types were also presented, with a bar plot illustrating their respective percentages. **C** The top 5 marker genes of all cells and the differentially expressed genes between the two groups were visualized using bubble plots. **D** GO-BP analysis was performed for all annotated cells. **E** GO-BP analysis was conducted for the upregulated and downregulated genes in cervical cancer fibroblasts. **F** GSEA analysis identified enriched pathways in fibroblasts. **G** An enrichment network diagram illustrated pathway analysis results based on fibroblast gene sets. **H** Fibroblasts were classified into six subtypes with distinct characteristics based on their marker genes. **I** The distribution of fibroblast subtypes between the Neoplasm group and the Normal tissue adjacent to neoplasm group was shown using UMAP plots, along with the distribution of cell cycle phases within each subtype. **J** Stacked bar plot depicted the percentage composition of fibroblast subtypes across different sample origins. **K** ST maps inferred the most likely cell types at selected points within cervical cancer tissue sections. **L** Differences in nFeature RNA, CNV score, and nCount RNA across fibroblast subtypes were demonstrated. **M** ST maps displayed nFeature RNA, CNV label, and nCount RNA for fibroblast subtypes. **N** A heatmap showcased differentially expressed marker genes across the six fibroblast subtypes. **O** GO-BP analysis was performed for the differentially expressed genes of each fibroblast subtype. **P** Word clouds highlighted terms strongly associated with each of the six fibroblast subtypes. **Q** ST maps visualized C0 MYH11 + fibroblasts. **R** GO-BP analysis identified upregulated genes in the Neoplasm group and the Normal tissue adjacent to neoplasm group within the C0 subtype. **S** GSEA analysis revealed enriched pathways for C0 MYH11 + fibroblasts

After batch correction was performed on all cells from the selected dataset, a total of 69,494 high-quality cells were obtained. These cells were classified into 9 cell types based on known marker genes, including endothelial cells (ECs), fibroblasts, epithelial cells (EPCs), smooth muscle cells (SMCs), B-plasma cells, mast cells, neutrophils, myeloid cells, and T-NK cells. It is important to note that before cell type annotation, tumor cells were identified by selecting epithelial cells based on their inferCNV results, highlighting those with high CNV scores (Supplementary Fig. 1 A). EPCs exhibited significantly higher G2/M and S scores compared to other cell types. EPCs, fibroblasts, ECs, myeloid cells, and SMCs had higher nCount RNA and nFeature RNA values (Fig. 2A).

The cells were categorized into two groups based on their origin: the Neoplasm group and the Normal tissue adjacent to neoplasm group. UMAP plots demonstrated that EPCs, neutrophils, myeloid cells, and T-NK cells were mainly concentrated in the Neoplasm group, while ECs, fibroblasts, and SMCs were predominantly located in the Normal tissue adjacent to neoplasm group. Bar plots indicated that EPCs constituted the largest proportion in the Neoplasm group, whereas fibroblasts were the dominant cell type in the Normal tissue adjacent to neoplasm group. Additionally, fibroblasts were primarily in the G1 phase, whereas EPCs were predominantly in the G2/M and S phases (Fig. 2B). Regarding differential gene expression, the top 5 marker genes for fibroblasts were PTGDS, DCN, COL1A1, COL1A2, and COL3A1. Genes with higher expression in the Normal tissue adjacent to neoplasm group included these markers as well as TAGLN and C11orf96 (Fig. 2C). GO-BP analysis of differentially expressed genes from each cell type revealed that fibroblasts were mainly enriched in processes such as external encapsulating structure organization, extracellular structure organization, and extracellular matrix organization (Fig. 2D). Further analysis showed that upregulated genes in fibroblasts were primarily associated with pathways like IL-17 signaling, legionellosis, lipid metabolism, atherosclerosis, TNF signaling, and antigen processing and presentation. Conversely, downregulated genes were linked to pathways related to the cytoskeleton in muscle cells, focal adhesion, vascular smooth muscle contraction, and proteoglycans in cancer (Fig. 2E). GSEA analysis highlighted two significant pathways for fibroblasts: the transmembrane receptor protein serine/threonine kinase signaling pathway and collagen fibril organization (Fig. 2F). The enrichment network diagram further revealed that gene sets across all fibroblast subtypes were most enriched in the cell surface receptor signaling pathway and serine hydrolase activity (Fig. 2G).

After re-performing batch correction, we classified 15,843 fibroblasts from the Neoplasm and Normal tissue adjacent to neoplasm groups into six distinct cell clusters. Before annotating the fibroblast subtypes, we observed the CNV (Copy Number Variation) patterns for each subtype (Supplementary Fig. 1B). We then named the subtypes based on their specific marker gene expression (Supplementary Fig. 2 A), and confirmed the expression of these six marker genes using spatial transcriptomics (Supplementary Fig. 2B). The six subtypes were as follows: C0 MYH11 + Fibroblasts, C1 CCN5 + Fibroblasts, C2 CXCL14 + Fibroblasts, C3 LUM + Fibroblasts, C4 GGT5 + Fibroblasts, and C5 ABCA8 + Fibroblasts (Fig. 2H). Except for the C2 subtype, which had some cells derived from the Neoplasm group, the majority of cells in the other subtypes originated from the Normal tissue adjacent to neoplasm group. Most cells in all fibroblast subtypes were in the G1 phase (Fig. 2I). Among the subtypes, C0 MYH11 + Fibroblasts showed the largest proportion in the three samples from the Normal tissue adjacent to neoplasm group (Fig. 2J). We also validated the presence of all fibroblast subtypes in the selected points of CC tissue sections using spatial transcriptomics, confirming the presence of all fibroblast subtypes (Fig. 2K, Supplementary Fig. 7 A).

We next visualized differences in nFeature RNA, CNV score, and nCount RNA across subtypes using UMAP plots. Notably, the C0 subtype exhibited elevated CNV scores (Fig. 2L). Deconvolution analysis of the C0 subtype revealed a higher abundance of cells from the Normal tissue adjacent to neoplasm group compared to those from the Neoplasm group. In addition to EPCs, the C0 subtype contained the second-highest proportion of cells, which was consistent with our observations. In GSE7803, GSE52903 and in-house bulk RNA-seq data, MYH11 expression was higher in adjacent normal tissues than in tumor tissues, which was consistent with single-cell analysis (Supplementary Figs. 2 C-E, Supplementary Fig. 7B). Spatial transcriptomics further indicated the potential presence of various CNV labels, with the CNV Label ST feature map showing labels 1–8, alongside normal labels (Fig. 2M). These CNV labels were associated with distinct CNV scores (Supplementary Fig. 2 F). We then examined differential gene expression across all fibroblast subtypes (Fig. 2N) and performed enrichment analyses on the differentially expressed genes for each subtype. GO-BP analysis revealed that the C0 subtype was primarily enriched in processes related to mRNA processing regulation and cytoplasmic translation (Fig. 2O). A word cloud for the C0 subtype highlighted key terms such as muscle, cardiac, depolymerization, differentiation, and growth (Fig. 2P). Based on spatial transcriptomics, we inferred the likely location of the C0 subtype within CC tissue sections, as depicted in Fig. 2Q. Furthermore, GO-BP analysis of upregulated genes in the Neoplasm and Normal tissue adjacent to neoplasm groups indicated that genes upregulated in the Normal tissue adjacent to neoplasm group were mainly enriched in pathways associated with coronavirus disease (COVID-19), lipid metabolism and atherosclerosis, IL-17 signaling, legionellosis, and TNF signaling (Fig. 2R). Finally, GSEA analysis of the C0 subtype revealed significant enrichment in muscle contraction, skeletal muscle organ development, and muscle system process (Fig. 2S).

### The stemness, metabolism, and CAF characteristics heterogeneity of fibroblast subtypes

**Fig. 3:**
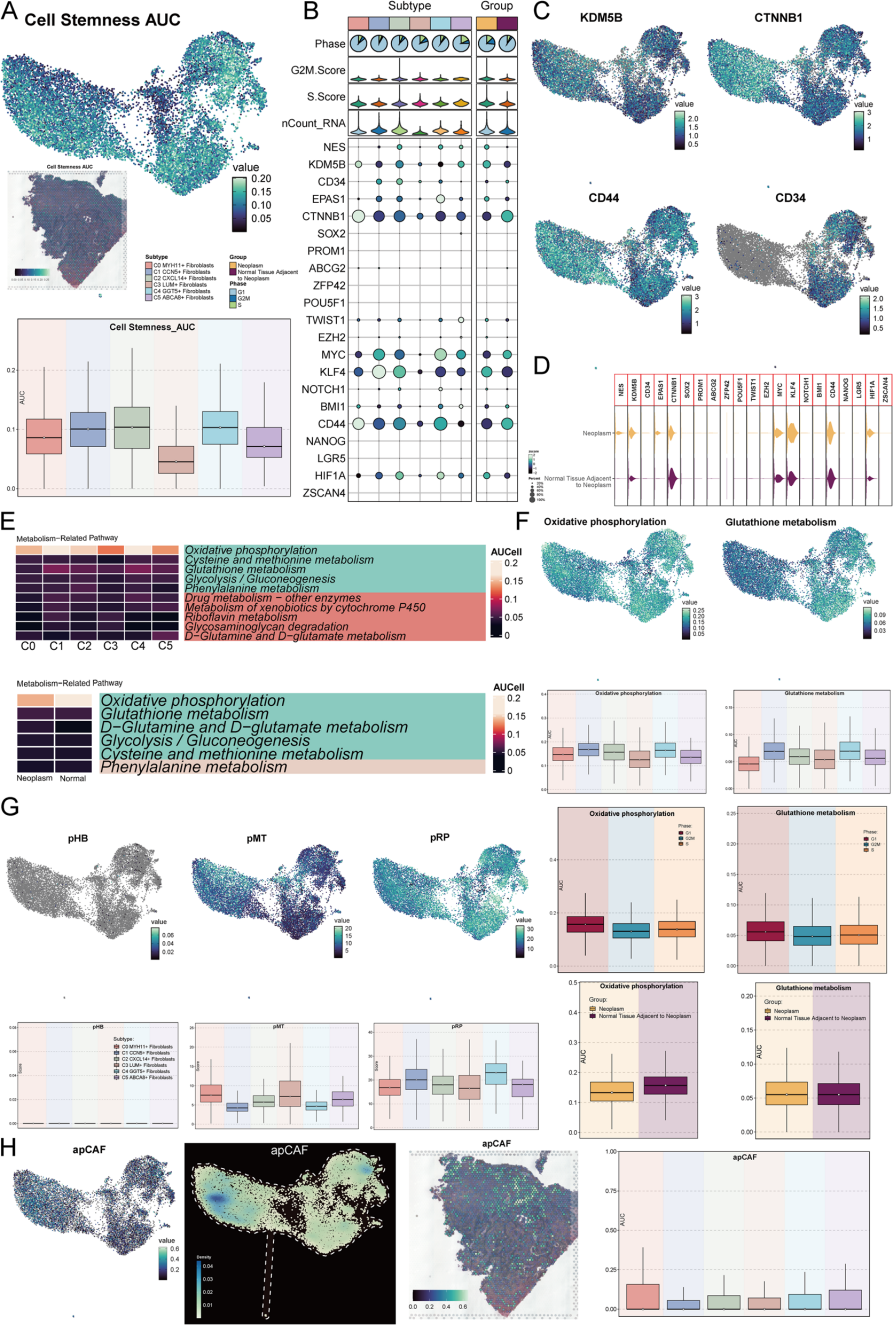
Scoring of Stemness, Metabolism, and apCAF Features in Fibroblast Subtypes. **A** The stemness of fibroblast subtypes was visualized using UMAP plots and ST feature maps. A boxplot illustrated the differences in stemness scores among subtypes. **B** A bubble plot displayed the expression levels of differentially expressed stemness-related genes across fibroblast subtypes. **C** UMAP plots highlighted the expression differences of stemness-related genes, including *KDM5B*, *CTNNB1*, *CD44*, and *CD34*, across fibroblast subtypes. **D** The differences in stemness-related gene expression between the Neoplasm group and the Normal tissue adjacent to neoplasm group were shown. **E** The significant metabolic pathways for each fibroblast subtype and for the two sample groups were identified and presented. **F** UMAP plots demonstrated differences in oxidative phosphorylation and glutathione metabolism across subtypes. Boxplots compared these two metabolic pathways across subtypes, cell cycle phases, and sample groups. **(G)** The differences in pHB, pMT, and pRP scores across fibroblast subtypes were visualized using UMAP plots and boxplots. **H** UMAP plots showed the differences in apCAF scores across subtypes in terms of expression and density. An ST feature map displayed apCAF scores spatially, and a boxplot quantitatively compared apCAF scores among fibroblast subtypes

To better understand the various differences between fibroblast subtypes and the specific characteristics of the key subtype, C0, we conducted a more comprehensive scoring and visualization analysis. First, we used UMAP to display the differences in cellular stemness across subtypes, and spatial transcriptomics analysis confirmed the maximum potential of the AUC values for cellular stemness. A box plot compared the stemness levels between subtypes, showing that the C2 subtype exhibited the highest stemness, followed by C1, C4, and C0 subtypes, while C3 had the lowest stemness (Fig. 3A). Next, a bubble plot visualized the expression differences of stemness genes between subtypes and between the two sample groups. The stemness genes highly expressed in the C0 subtype included CTNNB1, CD44, and KDM5B, while in the Normal tissue adjacent to neoplasm group, CTNNB1, CD44, and MYC were highly expressed (Fig. 3B). We then visualized the stemness genes highly expressed in the C0 subtype (KDM5B, CTNNB1, and CD44) using UMAP, contrasting with the stemness gene CD34, which was nearly absent in the C0 subtype (Fig. 3C). Furthermore, the violin plot was used to visually compare the expression levels of stemness genes between the two sample groups (Fig. 3D).

We then displayed the significant metabolic pathways for all fibroblast subtypes and the two sample groups using a heatmap (Fig. 3E). Both the C0 subtype and the Normal tissue adjacent to neoplasm group showed oxidative phosphorylation as the most prominent metabolic pathway, followed by glutathione metabolism. These two pathways were visualized specifically. Cells in both metabolic pathways were mostly in the G1 phase, but oxidative phosphorylation was more prominent in the Normal tissue adjacent to neoplasm group, while glutathione metabolism was less pronounced (Fig. 3F). We also scored the fibroblast subtypes for pHB, pMT, and pRP, and found that C0 had the highest pMT score compared to other subtypes, while its pRP score was the lowest. pHB was almost zero across all subtypes (Fig. 3G). Finally, to evaluate the CAF (Cancer-Associated Fibroblast) characteristics of the fibroblast subtypes in this dataset, we scored all subtypes for six related CAF traits. A bubble plot displayed the differences in CAF-related scores across the subtypes (Supplementary Fig. 3 A). We found that the C0 subtype had the most significant apCAF score difference compared to other subtypes. We then visualized the apCAF score, which was indeed higher in the C0 subtype, with a stronger density. A violin plot also confirmed this observation. Moreover, spatial transcriptomics validated the maximum potential of apCAF in CC tissue sections (Fig. 3H). The differences in scores and density for the other five CAF-related traits across the subtypes were also displayed through UMAP plots (Supplementary Figs. 3B, C), and further verified by spatial transcriptomics (Supplementary Fig. 3D). A box plot visually compared the levels of CAF-related scores across the subtypes (Supplementary Fig. 3E).

### Significant features of the C0 subtype in the pseudotime trajectory

**Fig. 4:**
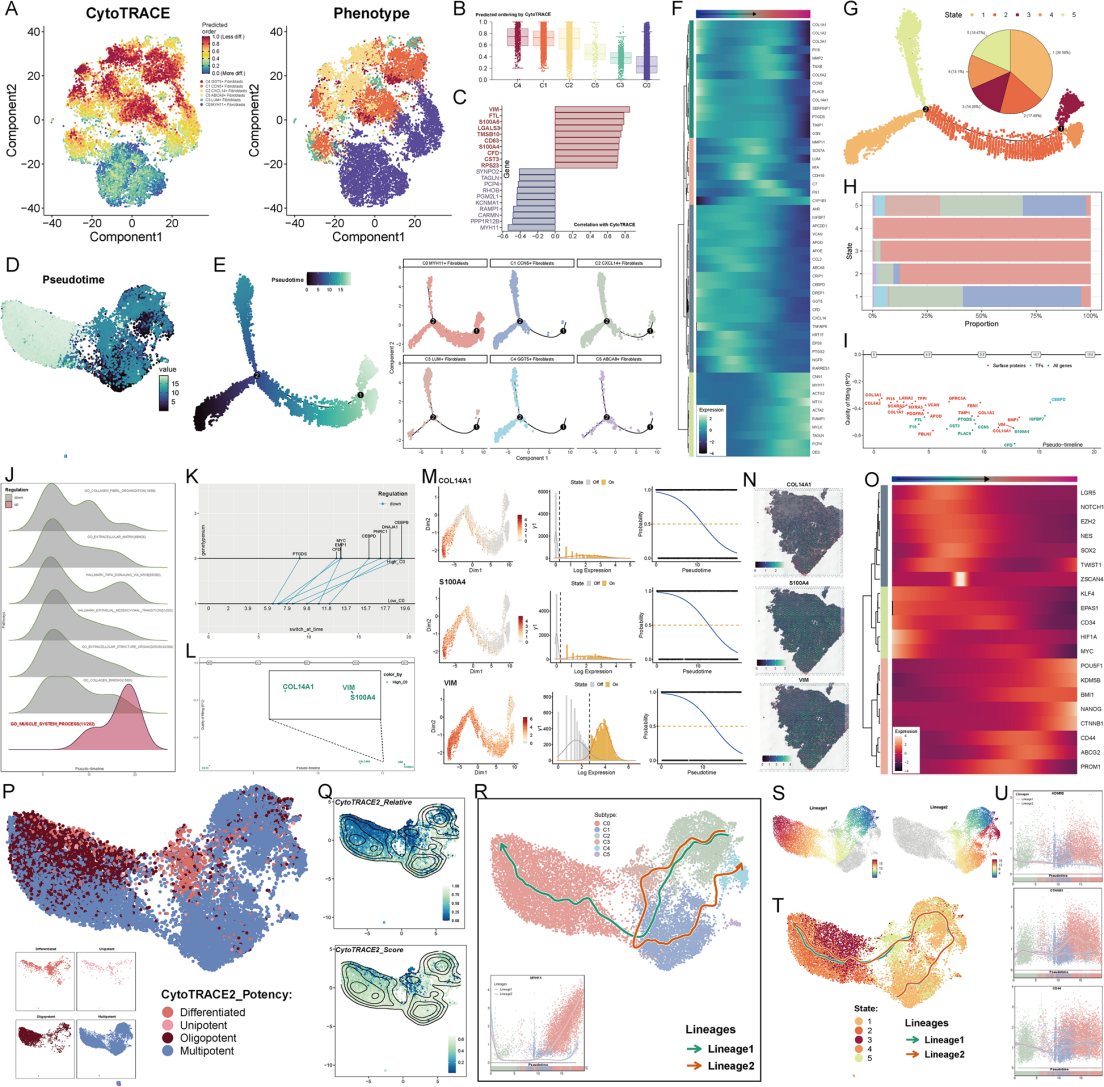
Pseudotime heterogeneity in fibroblast subtypes. **A**-**B** CytoTRACE analysis revealed the differentiation levels of the six fibroblast subtypes. **C** Genes positively and negatively correlated with CytoTRACE were identified. **D** UMAP plots indicated the primary pseudotime regions occupied by each subtype. **E** Pseudotime trajectory was constructed, progressing from left to right, showing the distribution of the six fibroblast subtypes along the trajectory. **F** A heatmap displayed the temporal expression patterns of differentially expressed genes across subtypes along the pseudotime trajectory. **G** The pseudotime trajectory was divided into five states (state1–5) based on branch points, with pie charts illustrating the percentage composition of each state. **H** Stacked bar plot depicted the proportional representation of each subtype within states 1–5. **I** GeneSwitches analysis demonstrated the distribution of surface proteins, transcription factors (TFs), and all genes along the pseudotime trajectory. **J** GO-BP analysis was performed for upregulated and downregulated genes at different time points along states 1–4 of the pseudotime trajectory. **K** Genes commonly expressed along both the High C0 and Low C0 pseudotime trajectories were identified. **L** Genes specifically expressed along the High C0 trajectory were highlighted, including *CST3* at the early stage and *COL14A1*, *VIM*, and *S100A4* at the late stage. **M** The expression patterns of *COL14A1*, *VIM*, and *S100A4* along the pseudotime trajectory were visualized. **N** ST feature maps depicted the spatial expression of *COL14A1*, *VIM*, and *S100A4*. **O** A heatmap illustrated the differential expression of stemness genes along the pseudotime trajectory. **P** CytoTRACE2 analysis revealed the expression distribution of stemness genes with varying efficacies. **Q** UMAP plots showed the relative density and scoring density of CytoTRACE2 across different subtypes. **R** Slingshot analysis constructed two trajectories, Lineage1 and Lineage2, among subtypes. The expression of the naming gene *MYH11* for C0 MYH11 + Fibroblasts was displayed along both trajectories. **S** UMAP plots illustrated the temporal progression of Lineage1 and Lineage2 across subtypes. **(T)** Based on the different states, the Slingshot trajectories (Lineage1 and Lineage2) were mapped to the distributions of each subtype. **U** Temporal changes in the expression of stemness-related genes (*KDM5B*, *CTNNB1*, and *CD44*) along Lineage1 and Lineage2 were visualized

First, CytoTRACE analysis revealed the differentiation levels of different fibroblast subtypes, with the C4 subtype showing the lowest differentiation and the C0 subtype the highest (Figs. 4A, B). Genes positively correlated with the CytoTRACE analysis include VIM, FTL, S100A6, LGALS3, TMSB10, CD63, S100A4, CFD, CST3, and RPS23 (Fig. 4C). Next, we displayed the distribution of pseudotime order across subtypes on a UMAP plot (Fig. 4D), and constructed a pseudotime trajectory with a time progression from left to right, featuring two branching points. We then mapped each fibroblast subtype onto this trajectory, with the majority of the C0 subtype positioned on the right branch (Fig. 4E). A heatmap shows the expression differences of differential genes as pseudotime progresses (Fig. 4F). To better define the positions on the pseudotime trajectory, we divided the entire trajectory into five parts based on the two branching points and named them five states. A pie chart displayed the proportion of each state in the entire pseudotime trajectory, with state1 accounting for the largest proportion at 36.58% (Fig. 4G). A stacked bar plot shows the proportion of each fibroblast subtype in each state, with the C0 subtype occupying the dominant position in states 2, 3, and 4 (Fig. 4H). Based on Monocle, we incorporated GeneSwitches, first presenting all surface proteins, transcription factors (TFs), and genes in the trajectory (Fig. 4I). Since we divided the trajectory into two main paths, one consisting of state1 and 5, and the other composed of states 1–4, we named the branch with more C0 subtype cells as High C0, while the other branch was named Low C0. We then performed a GO-BP analysis of the upregulated and downregulated genes along the High C0 trajectory, with the results shown in Fig. 4J. The upregulated genes were mainly enriched in the GO_MUSCLE_SYSTEM_PROCESS pathway. Next, we examined the heterogeneity between the High C0 and Low C0 trajectories. Common genes between the two trajectories included PTGDS, CFD, EMP1, MYC, CEBPD, PNRC1, DNAJA1, and CEBPB (Fig. 4K). Specific genes expressed in the High C0 trajectory included CST3, COL14A1, VIM, and S100A4 (Fig. 4L). Among these, CST3 was expressed early, while the other three genes were expressed later. Therefore, we selected COL14A1, S100A4, and VIM for visualization of their expression levels along the trajectory (Fig. 4M). Spatial transcriptomics confirmed the best possible presence of COL14A1, S100A4, and VIM (Fig. 4N).

Additionally, we specifically focused on the landscape of cell stemness and stemness gene expression along pseudotime. First, a heatmap displayed the temporal expression differences of stemness genes as pseudotime progressed (Fig. 4O). We also employed CytoTRACE2 to reveal the functional differences of stem cells across different fibroblast subtypes, with the UMAP plot showing that the C0 subtype mainly consists of pluripotent and oligopotent stem cells (Fig. 4P). To validate the accuracy of CytoTRACE2, we presented the relative density and scoring density of CytoTRACE2 across different subtypes on a UMAP plot (Fig. 4Q).

Finally, we used Slingshot to construct two trajectories, Lineage1 and Lineage2. In Lineage1, the developmental sequence was C2—C3—C1—C0, and the named gene MYH11 in the C0 subtype was highly expressed in the mid-to-late phase of both trajectories (Fig. 4R). The subsequent UMAP plot showed the range of fibroblast subtypes passed through by the two trajectories (Fig. 4S). Furthermore, a heatmap displayed the differential gene expression of the two trajectories, followed by an enrichment analysis. The results showed that genes highly expressed at the late stage of Lineage1 included CHRDL2, TAGLN, TPM2, CNN1, ACTC1, and TENM2. These differential genes were primarily enriched in terms like muscle, kidney, assembly, filopodium, myofibril, and cardiac (Supplementary Fig. 4 A). Next, two trajectories were constructed based on different states on the UMAP plot, and coincidentally, these two trajectories mostly overlapped, with their ends being at state2 and state4 where the C0 subtype was located (Fig. 4T). Finally, the stemness gene KDM5B was highly expressed in the late phase of Lineage1, CTNNB1 was also significantly upregulated at the late phase of Lineage1, and CD44 was highly expressed in the mid-to-late phase of both Lineage1 and Lineage2 (Fig. 4U).

### The C0 subtype interacts with tumor cells through the MDK-SDC1 pathway

**Fig. 5:**
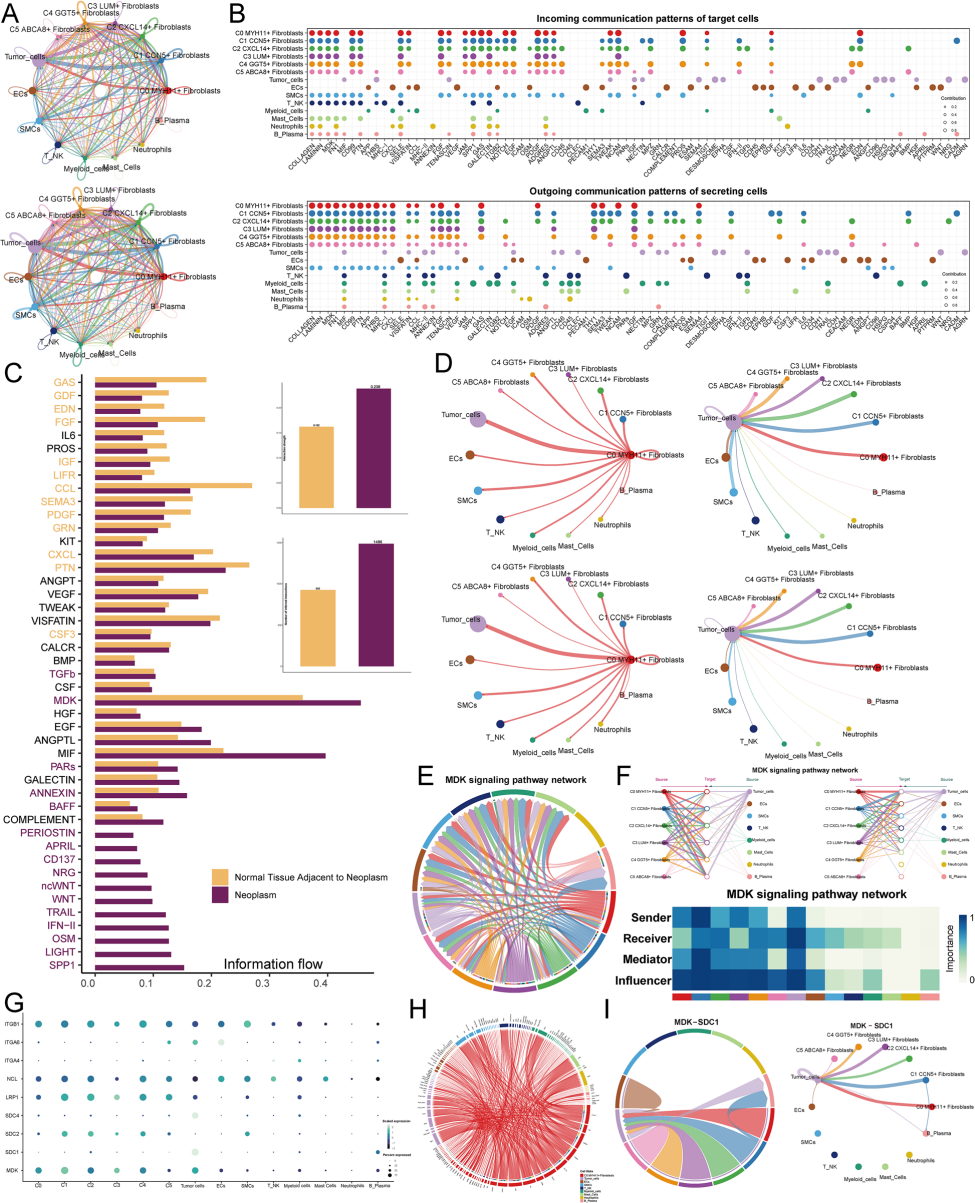
C0 MYH11 + Fibroblasts Interacted with Tumor Cells through MDK-SDC1 Crosstalk. **A** Circular plots illustrated the number (top) and strength (bottom) of interactions between all fibroblast subtypes and other cell types. **B** The bubble plots showed incoming communication patterns (top) of target cells and outgoing communication patterns (bottom) of secreting cells, respectively. **C** Differences in the signal intensity of pathways between the Neoplasm and Normal tissue adjacent to neoplasm groups were observed, along with a comparison of the number and strength of cell interactions in both groups. **D** Circular plots depicted the interaction number and intensity of C0 MYH11 + Fibroblasts with other cell types (left) and interactions from other cells to tumor cells (right). **E** Interaction networks among all cell types in the MDK signaling pathway were demonstrated. **F** The paracrine interactions of all cells in the MDK signaling pathway and the centrality scores of the MDK signaling pathway network. **G** Expression of various receptor proteins in different cell types along the MDK signaling pathway was shown. **H** A chord diagram displayed the interactions of C0 MYH11 + Fibroblasts with all other cell types. **I** Interactions among all cells in the MDK-SDC1 pathway were presented

To understand the interactions between the C0 subtype and tumor cells, we first displayed the interactions between all fibroblast subtypes and other cells using a circle plot (Fig. 5A). Both in terms of intensity and quantity, there were rich interactions between the fibroblast subtypes and various cells. Next, we particularly presented incoming communication patterns of target cells and outgoing communication patterns of secreting cells (Fig. 5B).The differential expression intensity of various signaling pathways between the Neoplasm and Normal tissue adjacent to neoplasm groups were showed (Fig. 5C). Notably, pathways such as TGFb, MDK, PARs, ANNEXIN, BAFF, PERIOSTIN, APRIL, CD37, NRG, TRAIL, IFN-II, OSM, etc, were significantly more expressed in the Neoplasm group (Fig. 5C). In both expression intensity and quantity, the Neoplasm group always showed higher values than the Normal tissue adjacent to neoplasm group. The interactions between the key subgroup of interest and tumor cells in CC are critical. To visualize this, we used a circle plot to display the number and intensity of interactions between the C0 subtype and other cell types (Fig. 5D). The results clearly showed that the C0 subtype interacted with a variety of cells, with significantly stronger and more frequent interactions observed with tumor cells. Additionally, another circle plot illustrated the interactions between all cell types and tumor cells, revealing that fibroblast subtypes had substantial interaction intensity and frequency with tumor cells. Next, we visualized the receptor-ligand pairs involved in C0 subtype-tumor cell interactions across both groups (Supplementary Fig. 4B). The MDK-related pathway stood out as particularly prominent, prompting us to use a chord diagram to illustrate all interactions within this pathway (Fig. 5E). We also drew a hierarchical diagram showing the autocrine and paracrine interactions among all cells in the MDK signaling pathway, thereby understanding that the C0 subtype is an important signal transduction transmitter, and tumor cells are its main receptors (Fig. 5F). And the analysis of the centrality scores of the MDK signaling pathway network indicated that the C0 subtype mainly functions as a sender, regulator, and influencer of the signal (Fig. 5F). To identify additional critical receptor proteins, we separately visualized the interactions between the C0 subtype and other cell types using bubble plots and chord diagrams (Fig. 5G, H). The MDK-SDC1 pathway emerged as the most significant, so we visualized all cell interactions within this pathway. Both the chord diagram and circle plot emphasized the MDK-SDC1 pathway as a key route for crosstalk between the C0 subtype and tumor cells (Fig. 5I). Moreover, spatial transcriptomics confirmed the prominent presence of SDC1 as the receptor protein (Supplementary Fig. 4 C).

### Transcription factor regulatory network landscape

**Fig. 6:**
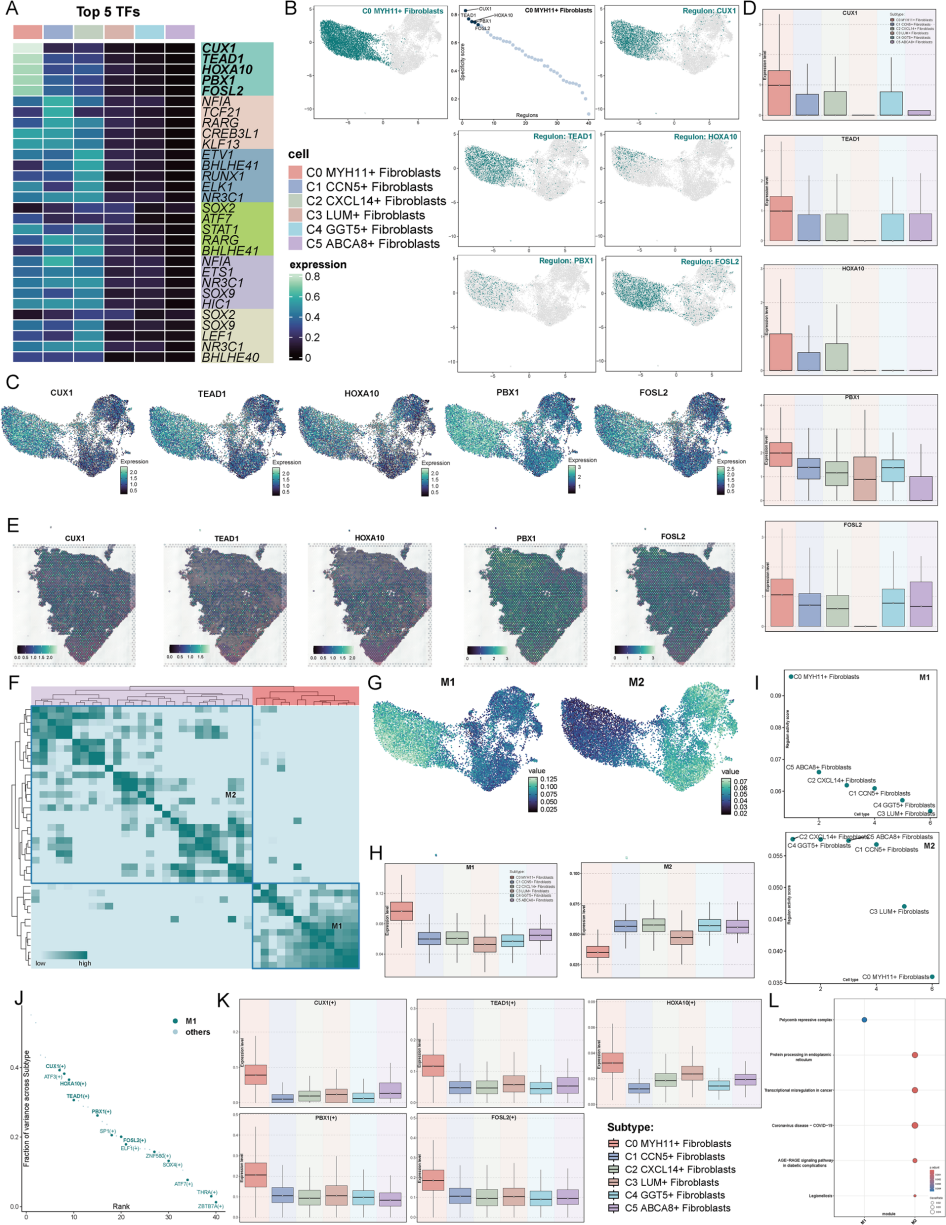
TFs Regulatory Network in Fibroblast Subtypes. **A** A heatmap displayed the differential expression of the top 5 transcription factors (TFs) across all fibroblast subtypes. **B** C0 MYH11 + Fibroblasts were highlighted with dark green dots on the UMAP plot (left). The ranking of regulatory factors in C0 MYH11 + Fibroblasts was shown based on the Regulatory Score (RSS) (middle), along with the expression distribution of the top 5 TFs in C0 MYH11 + Fibroblasts. **C** UMAP plots illustrated the expression intensity of the top 5 TFs in C0 MYH11 + Fibroblasts across various subtypes. **D** Boxplots visually compared the expression levels of the top 5 TFs across the different fibroblast subtypes. **E** ST feature maps displayed the expression of the top 5 TFs in C0 MYH11 + Fibroblasts. **F** A heatmap illustrated the similarity of regulatory submodules based on SCENIC recognition modules and AUCell scores. Submodules of regulators from fibroblast subtypes in both the Neoplasm and Normal tissue adjacent to neoplasm groups were identified, resulting in the identification of two submodules based on rule similarity. **G** UMAP plots showed the distribution of M1 and M2 modules across fibroblast subtypes. **H** The value of M1 and M2 modules was displayed. **I** The ranking of fibroblast subtype content within M1 and M2 modules was shown. **J** The ranking of TF expression intensity in M1 module was presented. **K** Differential expression levels of five TFs—*CUX1*, *TEAD1*, *HOXA10*, *PBX1*, and *FOSL2*—across different fibroblast subtypes were illustrated. **L** A bubble plot displayed the results of GO-BP analysis based on gene sets from M1 and M2 modules

To understand the transcription factors (TFs) differences among the subtypes, we attempted to visualize their transcriptional regulatory landscapes. First, we presented a heatmap showing the top 5 TFs for each subtype, where the top 5 TFs for the C0 subtype are CUX1, TEAD1, HOXA10, PBX1, and FOSL2 (Fig. 6A). Next, we ranked the regulatory factors of the C0 subtype according to the RSS and highlighted them with dark green dots in the UMAP plot. We also mapped the highest-regulated factors of the C0 subtype based on RAS on the UMAP, with their distribution shown by dark green dots, consistent with the heatmap results (Fig. 6B). UMAP plots showed that CUX1, TEAD1, HOXA10, PBX1, and FOSL2 are all more highly expressed in the C0 subtype (Fig. 6C), and boxplots provided a direct comparison (Fig. 6D). Spatial transcriptomics validated the possible presence of these 5 TFs (Fig. 6E).

We next applied the SCENIC identification rules and the CSI matrix to uncover the regulatory modules of fibroblast subtypes. Based on AUCell scores, we categorized the regulatory modules into two primary groups, M1 and M2 (Fig. 6F). When we visualized the average activity scores of each module on the UMAP, we observed distinct associations between the modules and different fibroblast subtypes (Fig. 6G). The M1 module of the C0 subgroup had the highest score, while the M2 module had the lowest score (Fig. 6H). Subsequently, we ranked the transcription factor (TF) activity scores for each subtype within M1 and M2. In M1, the C0 subtype showed significantly higher TF activity compared to the other subtypes, while in M2, C0 had markedly lower TF activity than the other subtypes (Fig. 6I). We also ranked the top 5 TFs in M1 based on the variance explained across subtypes, and these TFs were found to be among the highest-ranked (Fig. 6J). A comparison of the expression levels of these 5 TFs across subtypes revealed that all were significantly expressed in the C0 subtype (Fig. 6K). Finally, we conducted GO-BP analysis on the differentially expressed genes and TFs in M1 and M2. The results showed that M1 was predominantly enriched in polycomb repressive complex pathways, while M2 was primarily associated with processes such as protein processing in the endoplasmic reticulum, transcriptional misregulation in cancer, coronavirus disease (COVID-19), the AGE-RAGE signaling pathway in diabetic complications, and legionellosis (Fig. 6L).

### CC Prognostic Prediction Model Based on C0 Subtype

**Fig. 7:**
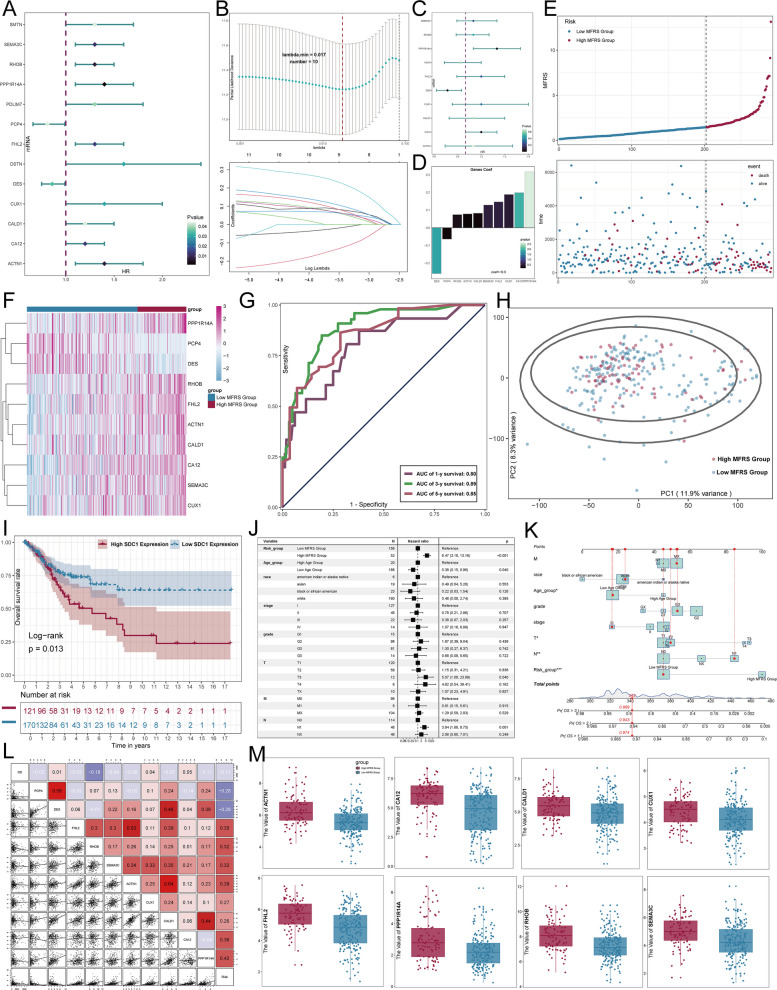
Key prognostic significance of C0 MYH11 + fibroblasts in cervical cancer patients. **A** A forest plot showed the results of univariate Cox analysis, identifying 13 C0 MYH11 + Fibroblasts-related genes associated with cervical cancer prognosis, with P ≤ 0.05. The reference line (HR = 1) distinguished protective factors (HR < 1) from risk factors (HR > 1). **B** Lasso regression was applied to select 10 genes contributing to the risk score, and the results were displayed in a lambda plot (lambda.min = 0.017). **C** A forest plot depicted the final 10 genes associated with cervical cancer prognosis. **D** A bar plot showed the coefficient values of the 10 risk genes. **E** A curve plot illustrated the risk scores in high MFRS and low MFRS groups, with scatter plots displaying survival/death events over time for the high MFRS and low MFRS groups. **F** A heatmap displayed the differential expression of the 10 risk genes between the high MFRS and low MFRS groups. **G** ROC curves showed the AUC values for 1-year (AUC = 0.80), 3-year (AUC = 0.89), and 5-year (AUC = 0.85) survival predictions. **H** A scatter plot displayed the distribution of genes along PC1 and PC2 for the high MFRS and low MFRS groups. **I** Kaplan–Meier survival curves showed survival differences between the high and low SDC1 expression groups. **J** A forest plot presented the results of multivariate Cox analysis for clinical factors and risk scores in the training cohort. **K** The nomogram model based on the MFRS was constructed, including race, age, and grade. **L** Scatter and heatmaps showed the correlation between the 10 prognostic genes and OS and MFRS. **M** Box and scatter plots illustrated the expression differences of 8 genes from the 10 risk genes between the high MFRS and low MFRS groups

Given the pivotal role of SDC1 in cervical cancer, we developed a prognostic model for CC based on the C0 subtype. Our analysis identified 10 genes that were significantly associated with patient prognosis (Fig. 7A-D). We then visualized survival and gene expression differences between the high MFRS group (MYH11 + Fibroblasts Risk Score) and the low MFRS group (Fig. 7E, F). The results demonstrated that the genes included in the model had strong predictive power (Fig. 7G, H). Moreover, the high SDC1 expression group showed a significantly lower overall survival rate compared to the low SDC1 expression group (*p* = 0.013) (Fig. 7I). To further evaluate the independent prognostic value of MFRS, we performed a multivariate Cox analysis for overall survival (OS) in the TCGA database (Fig. 7J). A nomogram that integrated the MFRS risk score, age categories (high and low), race (American Indian or Alaska Native, Asian, Black or African American, White), and tumor grade (G2, G3, GB, GX) was created to predict 1-, 2-, and 3-year OS in the training cohort (Fig. 7K). Additionally, we assessed and visualized the correlations between the 10 prognostic-related genes, OS, and MFRS using scatter plots and heatmaps (Fig. 7L). To further validate our findings, we randomly selected 8 genes from the 10 risk genes and compared their expression levels between the high and low MFRS groups. The results, presented in box plots and scatter plots, showed that all 8 genes were expressed at significantly higher levels in the high MFRS group compared to the low MFRS group (Fig. 7M).

### Immune infiltration analysis

To investigate the heterogeneity between the high MFRS and low MFRS groups, we performed an extensive analysis of their tumor immune microenvironment. Initially, a heatmap was generated to visualize the distribution of 22 distinct immune cell types in both groups (Supplementary Fig. 5 A). Boxplots revealed a higher representation of macrophages M0, CD4 memory resting T cells, and CD8 T cells. Furthermore, the high MFRS group showed a greater abundance of activated mast cells, macrophages M0, and resting NK cells compared to the low MFRS group (Supplementary Fig. 5B). Correlation analysis demonstrated a strong relationship between naive B cells and plasma cells, as well as between CD8 T cells and activated CD4 memory T cells (Supplementary Fig. 5 C). In addition, a positive correlation was found between risk score genes and MFRS groups with activated mast cells, macrophages M0, resting NK cells, neutrophils, resting CD4 memory T cells, gamma delta T cells, and activated NK cells (Supplementary Fig. 5D). We further utilized the CIBERSORT and Xcell algorithms to estimate the proportion of immune infiltrates in both MFRS groups (Supplementary Fig. 5E).

Next, we compared the StromalScore, ImmuneScore, and ESTIMATEScore between the groups, finding that all scores were higher in the low MFRS group (Supplementary Fig. 5 F). Tumor purity was greater in the high MFRS group, but no significant differences in tumor purity were observed between the high and low age groups. The TIDE score was slightly higher in the low MFRS group compared to the high MFRS group (Supplementary Fig. 5G-I).

Additionally, we assessed the somatic gene mutation frequencies in the training cohort and identified the top 20 genes with the highest mutation rates (Supplementary Fig. 5 J), with TTN showing the most frequent mutations. CNV analysis of the 10 prognostic-related genes revealed frequent gains and losses, particularly for PPP1R14 A, DES, and CALD1 (Supplementary Fig. 5 K). Tumor mutational burden (TMB) values were calculated for both groups, and their distribution was visualized using violin plots. Although no significant difference in TMB values was observed between the groups, the low MFRS group was more likely to exhibit high TMB (Supplementary Fig. 5L). Moreover, a negative correlation between TMB values and risk scores was observed (Supplementary Fig. 5 M), suggesting a potential relationship between these factors. Based on the median TMB value, the training cohort was divided into high and low TMB groups, which were further stratified by MFRS groups (Supplementary Figs. 5 N, O). The low TMB group had lower survival rates (Supplementary Fig. 5 N), and the high-risk-low TMB group had the lowest survival rate compared to the other three groups (Supplementary Fig. 5O).

### Tumor Border Characterization and Kaplan–Meier Survival Curves of Key Genes

**Fig. 8:**
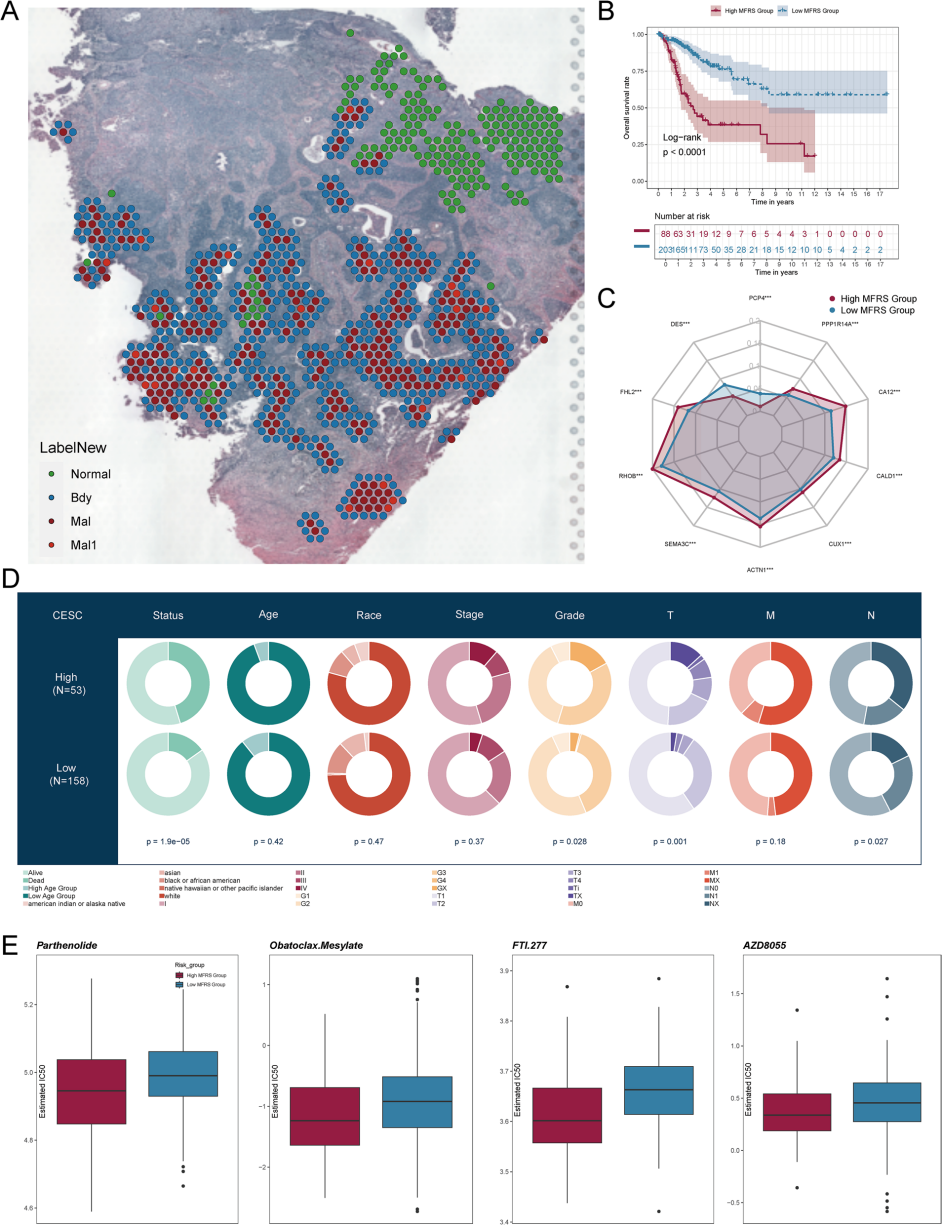
Tumor boundary characterization, prognostic model differences, and immune drug treatment prediction. **A** ST feature maps displayed the distribution of cervical cancer tumor boundaries (Bdy), malignant regions (Mal, Mal1), and normal tissue (Normal) in selected tissue sections. **B** Kaplan–Meier survival curves showed survival differences between the high MFRS and low MFRS groups. **C** Expression differences of 10 risk genes between the high MFRS and low MFRS groups were illustrated. **D** A pie chart displayed the proportions of different statuses, ages, races, stages, and grades in the study. **E** Boxplots showed the IC50 values of four immune drugs in the high MFRS and low MFRS groups

To begin, we continued utilizing spatial transcriptomics to generate the ST feature map shown in Fig. 8A, which provided a clearer depiction of the distribution of tumor borders, malignant regions, and normal tissues within the selected sections. Furthermore, when comparing the two groups, the high MFRS group exhibited a significantly lower overall survival rate than the low MFRS group (*p* < 0.0001) (Fig. 8B). We conducted survival curve analysis for key genes such as MYH11, KDM5B, CTNNB1, CD44, CUX1, TEAD1, HOXA10, PBX1, and FOSL2. With the exception of MYH11, all genes showed a lower survival rate in the high-expression group compared to the low-expression group (Supplementary Fig. 6 A).

We also visualized the expression patterns of the 10 prognostic genes, and similar to previous observations, only DES and PCP4 exhibited higher expression levels in the low MFRS group relative to the high MFRS group (Fig. 8C). Additionally, we analyzed the distribution of the prognostic genes across various status categories, such as age, race, stage, and grade (Fig. 8D). Finally, we examined the IC50 values of four immune-related drugs—Parthenolide, Obatoclax Mesylate, FTI.277, and AZD8055—between the high MFRS and low MFRS groups (Fig. 8E). The IC50 values of all four drugs were lower in the high MFRS group, while they were higher in the low MFRS group.

### Enrichment analysis between high and low MFRS groups

**Fig. 9:**
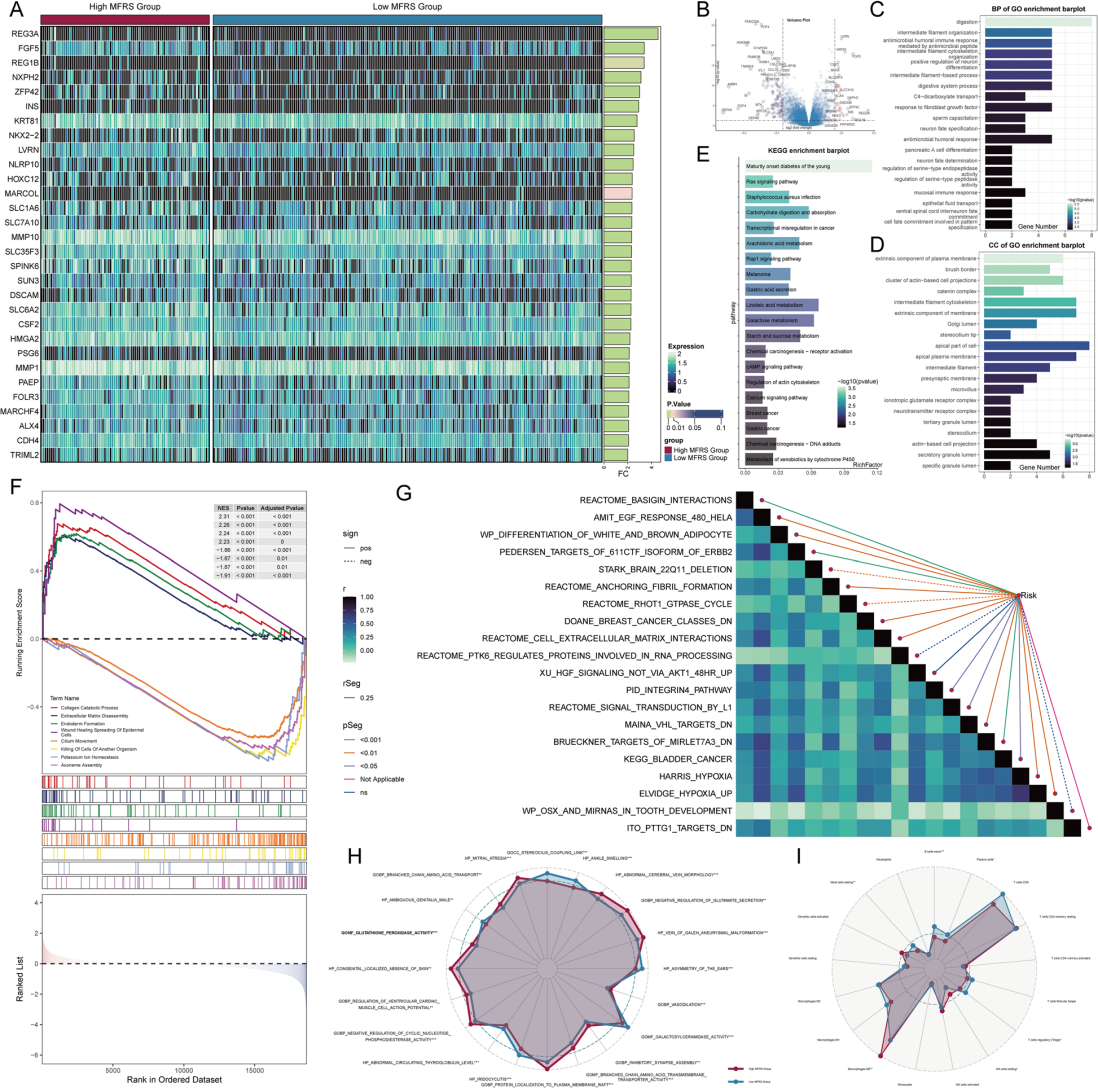
Enrichment analysis of differential genes in high and low MFRS Groups. **A** A heatmap displayed the expression profiles of differential genes between the high MFRS and low MFRS groups. **B** A volcano plot showed the differentially upregulated and downregulated genes in the high MFRS and low MFRS groups. **C-E** Bar plots presented the GO-BP, GO-CC, and KEGG analysis of differential genes in the high MFRS and low MFRS groups. **F** Detailed GSEA entries based on differentially upregulated and downregulated genes were presented. **G** A heatmap displayed the GSVA analysis of differential gene sets in the high MFRS and low MFRS groups. **H** A radar chart compared the differences in entries obtained through different enrichment methods between the high MFRS and low MFRS groups. **I** A radar chart showed the differences in the content of various immune cell types in different states between the high MFRS and low MFRS groups

To further explore the functional differences between the high MFRS and low MFRS groups, we analyzed the DEGs between the two groups and examined the pathways they were enriched in. Initially, we identified the DEGs between the high and low MFRS groups (Figs. 9A, B). We then conducted GO-BP, GO-CC, and KEGG pathway analyses (Figs. 9C-E). In the GO-BP analysis, the pathways were predominantly enriched in processes related to digestion, intermediate filament organization, and others. The GO-CC analysis revealed enrichment in the extrinsic component of the plasma membrane, brush border, clusters of actin-based cell projections, catenin complex, intermediate filament cytoskeleton, and extrinsic components of the membrane. KEGG pathway analysis highlighted pathways associated with maturity onset diabetes of the young, Ras signaling, Staphylococcus aureus infection, carbohydrate digestion and absorption, and transcriptional misregulation in cancer.

Additionally, we performed a GSEA analysis, which showed that the upregulated genes were primarily enriched in processes such as collagen catabolic processes, wound healing, epidermal cell spreading, endoderm formation, and extracellular matrix disassembly. In contrast, the downregulated genes were mainly enriched in potassium ion homeostasis, killing of cells from other organisms, axoneme assembly, and axoneme assembly (Fig. 9F).

We also correlated several of the enriched pathways with the risk scores from the prognostic model we constructed (Fig. 9G). Notably, terms such as PEDERSEN_TARGETS_OF_611 CTF_ISOFORM_OF_ERBB2,REACTOME_CELL_EXTRACELLULAR_MATRIX_INTERACTIONS,BRUECKNER_TARGETS_OF_MIRLET7 A3_DN, and ELVIDGE_HYPOXIA_UP showed a significant positive correlation with the risk score, suggesting potential statistical significance. To further investigate significant pathways enriched in the high and low MFRS groups, we used a radar chart, which revealed that terms like GOBP_BRANCHED_CHAIN_AMINO_ACID_TRANSPORT, GOBP_REGULATION_OF_VENTRICULAR_CARDIAC_MUSCLE_CELL_ACTION_POTENTIAL,GOBP_NEGATIVE_REGULATION_OF_CYCLIC_NUCLEOTIDE_PHOSPHODIESTERASE_ACTIVITY,GOBP_PROTEIN_LOCALIZATION_TO_PLASMA_MEMBRANE_RAFT,GOBP_INHIBITORY_SYNAPSE_ASSEMBLY,andGOBP_NEGATIVE_REGULATION_OF_GLUTAMATE_SECRETION were significantly enriched in the high MFRS group (Fig. 9H).

Finally, to explore the immune cell landscape in both groups, we found that activated dendritic cells, M0 macrophages, activated NK cells, and resting NK cells were more abundant in the high MFRS group (Fig. 9I).

### SDC1 promotes proliferation, migration, and invasion of CC Cells

**Fig. 10:**
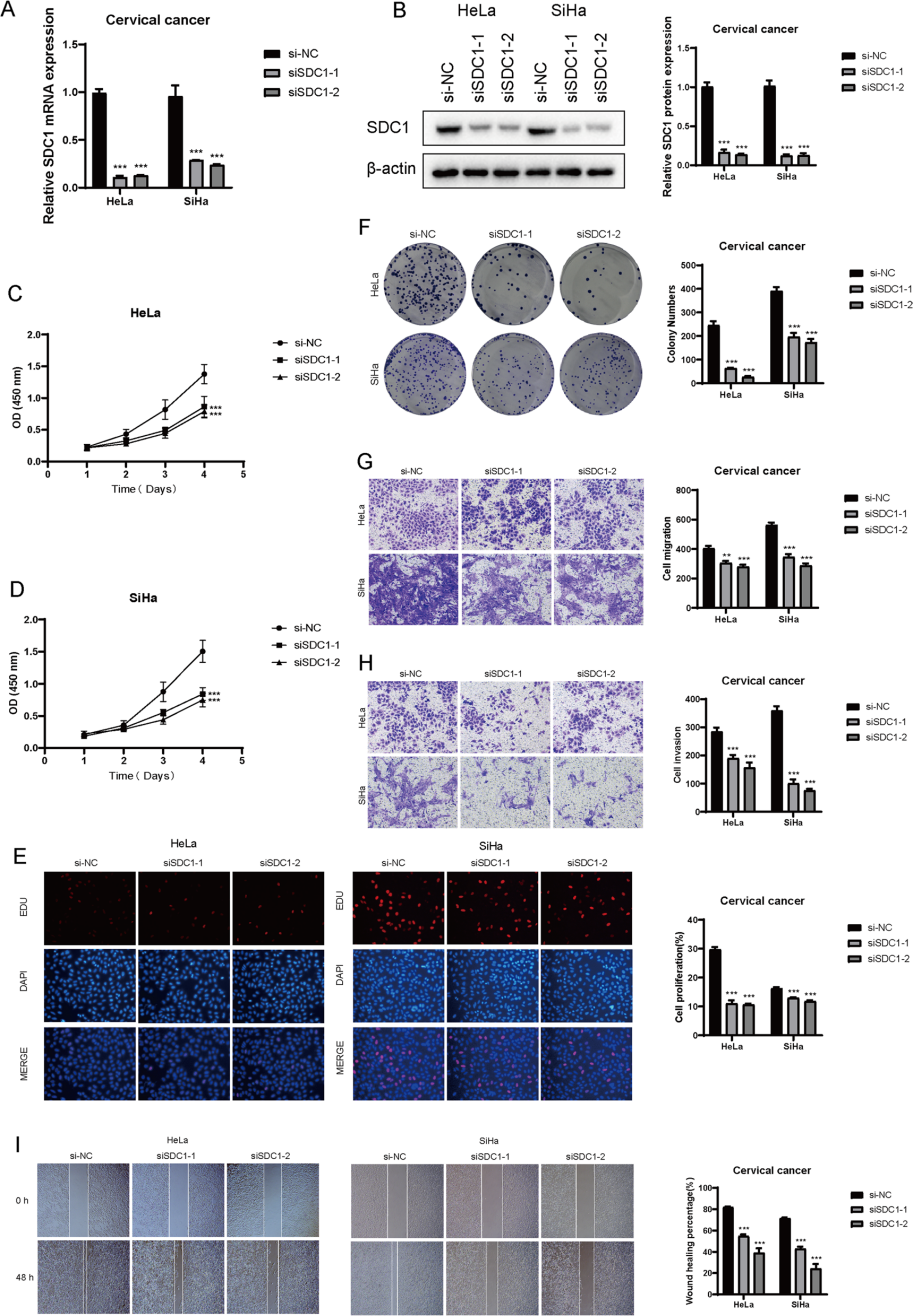
Knockdown of SDC1 Inhibits Proliferation, Migration, and Invasion of Cervical Cancer Cells. **A** The relative expression of SDC1 mRNA in cervical cancer cells was measured after knockdown in HeLa and SiHa cells. **B** The relative expression of SDC1 protein in HeLa and SiHa cells following SDC1 knockdown was evaluated. **C-D** CCK-8 assays showed a significant decrease in cell viability in HeLa and SiHa cells after SDC1 knockdown. **E** EDU staining experiments indicated that downregulation of SDC1 hindered cell proliferation in both HeLa and SiHa cells. **F** Colony formation assays demonstrated that cells with reduced SDC1 expression formed significantly fewer colonies compared to the NC group. **G-H** Transwell assays revealed that SDC1 knockdown significantly reduced the migration and invasion of cervical cancer cells. **I** Scratch wound healing assays showed that reduced SDC1 expression significantly decreased the wound healing rate. *P < 0.01, **P < 0.001, ***P < 0.0001, and ****P < 0.00001

To investigate the specific role of SDC1 in CC, we performed in vitro experiments. We knocked down SDC1 in two CC cell lines, HeLa and SiHa, and observed a significant reduction in the relative expression of SDC1 mRNA (Fig. 10A). Similarly, SDC1 protein expression was also notably downregulated after knockdown (Fig. 10B). CCK-8 assays showed that knocking down SDC1 resulted in a significant decrease in cell viability in both HeLa and SiHa cells (Figs. 10C, D). EDU staining experiments indicated that knocking down SDC1 significantly reduced the proliferative capacity of CC cells (Fig. 10E). Colony formation assays demonstrated that the number of cell colonies formed after SDC1 knockdown was significantly lower than the control group, further confirming the reduced proliferative ability (Fig. 10F). Finally, both Transwell and wound healing assays showed that the reduced expression of SDC1 significantly impaired the migration and invasion abilities of CC cells (Figs. 10G-I).

### Characterization of Fibroblast Heterogeneity and Spatial Distribution

To investigate the heterogeneity and spatial organization of fibroblasts within the tumor microenvironment, multiplex immunohistochemistry (mIHC) was performed on formalin-fixed paraffin-embedded (FFPE) human tissue samples from the tumor zone (TZ), junctional zone (JZ), and normal zone (NZ), as well as on isolated normal fibroblasts (NFs) and cancer-associated fibroblasts (CAFs). A panel of fibroblast-related markers, including α-smooth muscle actin (α-SMA), Vimentin, Calponin, Fibronectin, and myosin heavy chain 11 (MYH11), was evaluated.

**Fig. 11:**
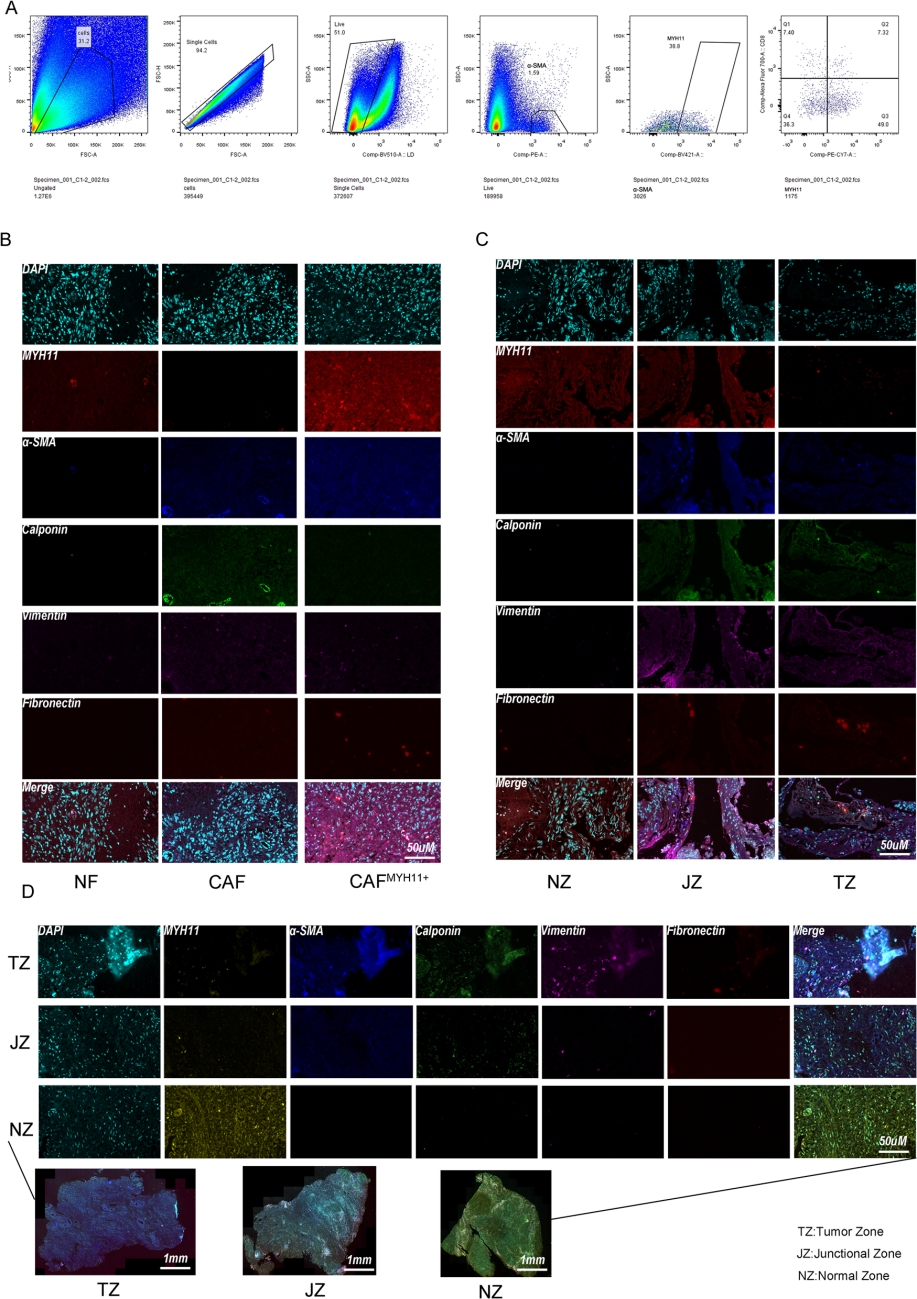
Characterization of fibroblast heterogeneity and spatial distribution. **A** Flow cytometric gating strategy identifying live, single CD45⁻CD31⁻α-SMA⁺ fibroblasts and MYH11-defined subpopulations from isolated normal fibroblasts (NFs) and cancer-associated fibroblasts (CAFs). **B** Comparative expression of fibroblast markers showing higher MYH11 expression in NFs, whereas CAFs exhibit increased Calponin, Vimentin, and Fibronectin levels. **C** Spatial distribution of fibroblast activation markers across normal zone (NZ), junctional zone (JZ), and tumor zone (TZ), revealing a progressive increase in α-SMA, Calponin, Vimentin, and Fibronectin, and a reciprocal decrease in MYH11. **D **Representative low- and high-magnification mIHC images demonstrating zonal co-localization patterns: α-SMA, Calponin, Vimentin, and Fibronectin are predominantly expressed in TZ, partially overlapping in JZ, and minimally detected in NZ, while MYH11 is enriched in NZ with reduced expression in JZ and absent in TZ

Flow cytometry was employed to define live, single CD45⁻CD31⁻α-SMA⁺ fibroblast populations (Fig. 11A), with MYH11 used to further discriminate subpopulations. MYH11 expression was found to be substantially higher in NFs compared to CAFs. Conversely, CAFs exhibited elevated expression of Calponin, Vimentin, and Fibronectin, which were minimally or not expressed in NFs (Fig. 11B).

Spatial analysis of tissue sections revealed a progressive increase in fibroblast activation marker expression from NZ to TZ (Fig. 11C). Specifically, α-SMA, Calponin, Vimentin, and Fibronectin were weakly expressed in NZ, moderately expressed in JZ, and strongly co-expressed in TZ. In contrast, MYH11 demonstrated an opposing gradient, with robust expression in NZ, moderate levels in JZ, and minimal expression in TZ. These findings suggest a spatially regulated fibroblast activation trajectory along the NZ–JZ–TZ axis.

High- and low-magnification mIHC images (Fig. 11D) confirmed the co-localization of α-SMA, Calponin, Vimentin, and Fibronectin predominantly in TZ, with partial overlap observed in JZ and minimal expression in NZ. Conversely, MYH11 co-localized primarily in NZ, with reduced expression in JZ and negligible signal in TZ. Collectively, these data reveal a spatially distinct fibroblast activation pattern, underscoring the phenotypic heterogeneity and dynamic stromal remodeling within the tumor microenvironment.

### C0 MYH11 + CAF Promotes tumor cell proliferation, migration, and inhibits apoptosis via soluble SDC1

**Fig. 12:**
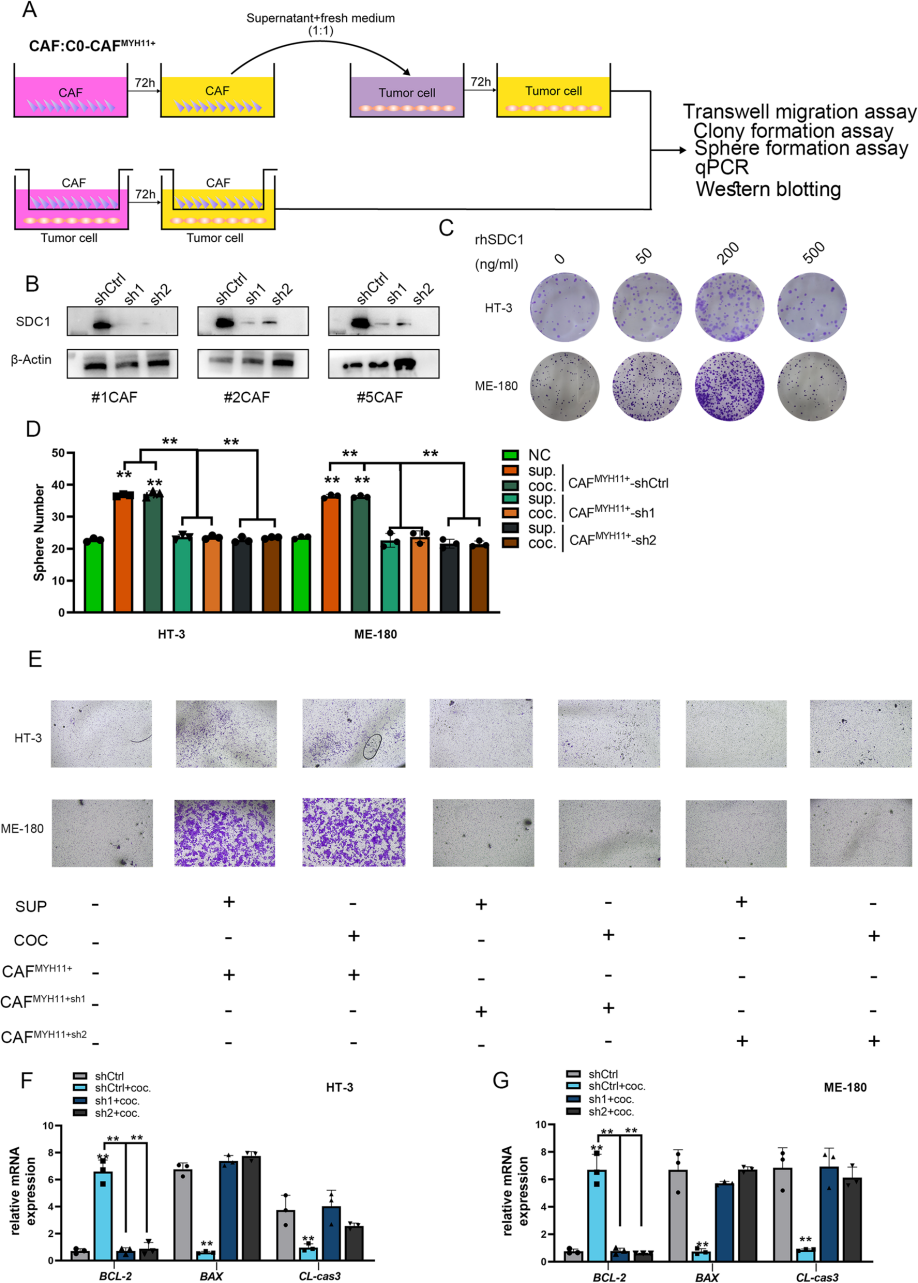
C0 MYH11 + CAF promotes tumor cell proliferation, migration, and inhibits apoptosis via soluble SDC1. **A** Experimental procedure for co-culture of C0 MYH11 + CAF with high- and low-invasive cervical cancer cells. Supernatants were collected and used to treat tumor cells. **B** shRNA-mediated silencing of SDC1 in C0 MYH11 + CAF resulted in reduced SDC1 expression. Western blot showing SDC1 levels in control and silenced groups. **C** Recombinant SDC1 treatment (200 ng/mL) promoted tumor cell proliferation, whereas 500 ng/mL inhibited proliferation. **D** Spheroid formation assay showing enhanced stemness and malignancy in tumor cells co-cultured with C0 MYH11 + CAF or treated with its supernatant. **E** Migration assay indicating increased migratory ability of tumor cells exposed to C0 MYH11 + CAF or its culture supernatant. **F**-**G** qRT-PCR analysis of apoptosis-related gene expression showing upregulation of anti-apoptotic and downregulation of pro-apoptotic genes in tumor cells treated with C0 MYH11 + CAF supernatant, suggesting reduced apoptosis

To further elucidate the functional characteristics of C0 MYH11 + CAF, we co-cultured isolated C0 MYH11 + CAF with high- and low-invasive cervical cancer cells. The experimental procedure is outlined in Fig. 12A, where fibroblasts were co-cultured with tumor cells, and the culture supernatant was subsequently collected to treat the tumor cells. To investigate the role of SDC1, we performed siRNA-mediated silencing of the SDC1 gene in C0 MYH11 + CAF, resulting in a significant reduction in SDC1 protein expression, as shown in Fig. 12B. Next, we assessed the optimal concentration of recombinant human SDC1 protein to investigate the effect of soluble SDC1 on tumor cells. As shown in Fig. 12C, treatment with 200 ng/mL of SDC1 led to the strongest promotion of tumor cell proliferation, while a concentration of 500 ng/mL suppressed cell proliferation, likely due to cytotoxic effects.

We then performed spheroid formation assays to evaluate the impact of C0 MYH11 + CAF on the stemness of tumor cells. As shown in Fig. 12D, tumor cells treated with the culture supernatant of C0 MYH11 + CAF or co-cultured with C0 MYH11 + CAF formed significantly more spheroids, demonstrating enhanced stemness and increased malignancy. Migration assays were conducted, and the results in Fig. 12E indicate that tumor cells exposed to C0 MYH11 + CAF or its culture supernatant exhibited a significant increase in migration, suggesting enhanced invasive ability.

Furthermore, qRT-PCR analysis revealed that tumor cells co-cultured with C0 MYH11 + CAF or treated with its culture supernatant showed significant upregulation of anti-apoptotic gene mRNA and downregulation of pro-apoptotic gene mRNA, leading to reduced apoptosis (Figs. 12F-G). These results collectively suggest that C0 MYH11 + CAF promotes tumor cell proliferation, migration, and stemness, while inhibiting apoptosis, potentially through the action of soluble SDC1.

## Discussion

CC ranks as the fourth most prevalent cancer impacting women's health globally [19, 20]. Approximately 70–80% of CC cases are squamous cell carcinomas, 20–25% are adenocarcinomas, and the remaining cases consist of adenosquamous carcinomas and rare histological types [21, 22]. Most cases of CC are caused by genital infection with human papillomavirus (HPV) [23]. Primary CC patients who undergo radical hysterectomy typically have a good prognosis, but the 5-year overall survival rate and disease-free survival rate for advanced-stage CC are relatively low [24, 25]. Exploring the pathogenesis of CC and discovering new therapeutic targets are crucial for enhancing patient survival rates. Research has indicated that the extent of fibroblast infiltration is linked to a poorer prognosis in CC [26], and the fibroblast growth factor receptor (FGFR) axis plays a role in CC progression [27–30]. Among the cell types that constitute the tumor microenvironment (TME), cancer-associated fibroblasts (CAFs) are key factors influencing phenotypic heterogeneity. CAFs are often derived from activated normal fibroblasts and can enhance tumor proliferation, invasion, and metastasis [31–35]. However, the gene expression patterns and functions of fibroblasts may vary across different subtypes and tumor types, further complicating the TME. Therefore, further exploration of the characteristics of fibroblasts in CC clinical samples is needed. Moreover, previous studies have shown that CC also has certain specificity in TME [36, 37].

In this study, we categorized cells into the Neoplasm group and the Normal tissue adjacent to neoplasm group based on the source of the samples from the cervical cancer dataset. We found that fibroblasts were most abundant in the Normal tissue adjacent to neoplasm group. We then analyzed the differential genes of fibroblasts, identifying the top five marker genes as PTGDS, DCN, COL1 A1, COL1 A2, and COL3 A1. Studies have shown that PTGDS exhibits abnormal expression and methylation in CC, suggesting its high prognostic value [38]. DCN is downregulated in CC tissues and cells, playing a key role in the development and progression of CC [39]. COL1 A1 facilitates the proliferation and metastasis of CC cells [40], and its expression level is negatively correlated with the radiosensitivity of CC patients. Activation of COL1 A1 can prevent apoptosis in CC cells [41]. Among these differential genes, upregulated genes were mainly enriched in pathways such as the IL-17 signaling pathway, legionellosis, lipid and atherosclerosis, TNF signaling pathway, and antigen processing and presentation. Interleukin-17 (IL-17) is a pro-inflammatory cytokine that plays an important role in inflammation, autoimmunity, and cancer. Studies have shown that IL-17 can indirectly regulate macrophage differentiation by upregulating cyclooxygenase-2 (COX-2) expression in the HeLa CC cell line, thereby modulating the tumor immune microenvironment [42]. IL-17 activates the JAK2/STAT3 and PI3 K/Akt/NF-κB signaling pathways to promote the progression of CC [43]. Increasing evidence indicates that genes from the tumor necrosis factor (TNF) family are critical in CC, potentially serving as prognostic biomarkers and being associated with responses to immunotherapy [44–47]. Given the correlation between HPV infection and the development of CC, investigating the antigen processing and presentation processes in the immune microenvironment is crucial for exploring the role of fibroblasts in the CC immune landscape. Next, we classified fibroblasts into six subtypes: C0 MYH11 + Fibroblasts, C1 CCN5 + Fibroblasts, C2 CXCL14 + Fibroblasts, C3 LUM + Fibroblasts, C4 GGT5 + Fibroblasts, and C5 ABCA8 + Fibroblasts, and further annotated these subtypes. We observed that the C0 subtype exhibited a higher CNV score, with its differential genes primarily enriched in the regulation of mRNA processing and cytoplasmic translation. GSEA analysis revealed enrichment in terms related to muscle contraction, skeletal muscle organ development, and muscle system processes.

We conducted a more comprehensive scoring and visualization of the key C0 subtype. In this subtype, the stemness-related genes with higher expression were CTNNB1, CD44, and KDM5B. CTNNB1 is a crucial gene in the Wnt/β-catenin signaling pathway and has etiological significance in CC models. Studies have suggested that genetic variations in CTNNB1 may contribute to the development of CC [48]. CD44 is a cell surface molecule, and CD44 + CC cells can resist radiation-induced apoptosis, exhibiting stem cell-like properties [49]. KDM5 encodes lysine-specific histone demethylases that are involved in maintaining genome stability and facilitating DNA repair. Research has demonstrated that KDM5B is overexpressed in CC tissues and cells, and silencing its expression can inhibit the growth of CC cells [50, 51]. Additionally, KDM5B transcriptionally activates AUP1 to reprogram lipid metabolism, thus promoting CC progression [52]. The most prominent metabolic pathway in the C0 fibroblast subtype is oxidative phosphorylation. The pMT score in this subtype was higher compared to other subtypes, while the pRP score was the lowest. The apCAF score showed more significant differences between subtypes, and spatial transcriptomics confirmed the highest likelihood of apCAF in CC tissue slices.

Next, we performed CytoTRACE analysis to reveal the differentiation degrees of different fibroblast subtypes and inferred that the C4 subtype has the lowest degree of differentiation, while the C0 subtype has the highest. In the pseudotime trajectory analysis, the C0 subtype showed a dominant presence in states 2, 3, and 4. Genes specifically expressed in the high C0 subtype included CST3, COL14 A1, VIM, and S100 A4. We visualized the expression levels of COL14 A1, S100 A4, and VIM along the trajectory, and spatial transcriptomics confirmed the highest likelihood of these genes'presence. We further used CytoTRACE2 to assess the stemness cell efficacy across the different fibroblast subtypes. The UMAP plot showed that the C0 subtype mainly consists of pluripotent and oligopotent stem cells. Finally, we employed Slingshot to construct two trajectories, Lineage1 and Lineage2. The findings revealed that the key C0 gene MYH11 was highly expressed during the mid- to late stages of both trajectories. Subsequently, we constructed two trajectories on the UMAP plot based on different states. Interestingly, these two trajectories largely overlapped, with their endpoints corresponding to states 2 and 4, where the C0 subtype predominantly resides.

For the C0 subtype, we focused on its interaction with tumor cells in CC. The chord diagram shows that the C0 subtype is connected to other cell types, with a particularly significant intensity and quantity of interaction with tumor cells. In the subsequent receptor-ligand visualization analysis, we found that the MDK-related pathway was particularly prominent, functioning as a ligand interacting with tumor cells. Through the visualization of C0 subtype interactions with other cells, we identified the MDK-SDC1 interaction pattern, which is an indispensable pathway for the crosstalk between C0 subtype and tumor cells. Midkine (MDK) is a promising tumor marker. Functional studies have shown that MDK interacts with Syndecan-1 (SDC1) through autocrine and paracrine mechanisms, activating the PI3 K/AKT and p38 MAPK pathways, enhancing CC cell proliferation, migration, and invasion, promoting lymphangiogenesis, and downregulating tight junction proteins in human lymphatic endothelial cells (HLECs) to facilitate lymph node metastasis [53]. SDC1 acts as a receptor tyrosine kinase and a matrix receptor, serving as a co-receptor for various signaling pathways. It regulates cell proliferation, adhesion, and migration, and is regarded as a key modulator of the tumor microenvironment [54]. It can function as a paracrine effector. Syndecans, including SDC1, exhibit variable expression in several solid tumors and hematologic cancers, with high expression of SDC1 in the cytoplasm of tumor cells correlating with better patient survival [55]. SDC1 regulates CC cell proliferation, the cell cycle, and apoptosis, with overexpression of SDC1 and its soluble form increasing CC invasiveness [56]. Additionally, SDC1 is linked to immune cell infiltration in CC [57, 58]. Therefore, exploring the MDK-SDC1 interaction pattern is crucial for understanding the pathogenesis and progression of CC.

Given the pivotal role of SDC1 in CC patients, we developed a prognostic model for CC based on the C0 subtype. The high SDC1 expression group showed a significantly lower overall survival rate compared to the low SDC1 expression group. Furthermore, we visualized the survival and gene expression differences between the high MFRS group (MYH11 + Fibroblasts risk score) and the low MFRS group, while assessing the independent predictive power of MFRS for overall survival (OS) using the TCGA database. The findings highlighted that MFRS could serve as a robust prognostic model for CC with strong predictive capabilities. The high MFRS group exhibited a markedly lower overall survival rate than the low MFRS group. Lastly, we evaluated the IC50 values of four immune drugs—Parthenolide, Obatoclax Mesylate, FTI.277, and AZD8055—which showed lower IC50 values in the high MFRS group and higher values in the low MFRS group, suggesting a better prediction of immune therapy response in the high MFRS group.

We also performed a comprehensive analysis of the tumor immune microenvironment in the high MFRS group and low MFRS group. Macrophages M0, T cells CD4 memory resting, and T cells CD8 were highly represented in both groups. Furthermore, Mast cells activated, macrophages M0, NK cells resting, Neutrophils, T cells CD4 memory resting, T cells gamma delta, and NK cells activated showed a positive correlation with risk score genes and both high and low MFRS groups. Yin's study found that MYH11 is a central gene in acute aortic dissection and, according to immune infiltration analysis, MYH11 is associated with macrophages M2 [59]. Another study on thyroid eye disease indicated that MYH11, along with CD4/CD8 T cells and B cells, was highly expressed in the periorbital fat tissue of patients and played a key role in disease progression [60]. MYH11 is also a prognostic gene in head and neck squamous cell carcinoma, with significantly reduced infiltration of CD8 + T cells, B cells, neutrophils, and NK cells in high-risk groups [61]. These pieces of evidence further confirm the correlation between MFRS, tumor immunity, and immune cell infiltration.

The differential gene expression enrichment analysis between the high MFRS group and low MFRS group identified several key enriched pathways, including maturity onset diabetes of the young, Ras signaling, Staphylococcus aureus infection, carbohydrate digestion and absorption, and transcriptional misregulation in cancer. In addition, pathways such as branched-chain amino acid transport, regulation of ventricular cardiac muscle cell action potential, negative regulation of cyclic nucleotide phosphodiesterase activity, protein localization to the plasma membrane raft, inhibitory synapse assembly, and negative regulation of glutamate secretion were notably more prominent in the high MFRS group. To examine the immune cell landscape in both MFRS groups, we observed a higher prevalence of immune cells, such as activated dendritic cells, macrophages M0, activated NK cells, and resting NK cells, in the high MFRS group. These results suggest a potential link between MFRS and immune cell activation, highlighting its role in immune modulation within the tumor microenvironment.

For the transcription factor regulation of the C0 subtype, we attempted to visualize its transcriptional regulatory landscape. The top five transcription factors (TFs) for the C0 subtype were CUX1, TEAD1, HOXA10, PBX1, and FOSL2, which are significantly expressed in this subtype. Spatial transcriptomics also confirmed the potential presence of these five TFs. Other studies have provided further insights into their roles: CUX1 has been identified as a methylation-associated differential gene in CC, with prognostic value and the potential to predict responses to immunotherapy [62]. TEAD1 can bind to and activate the long control region (LCR) of human papillomavirus (HPV) oncogenes E6 and E7, enhancing the expression of HPV oncogenes. It also interacts with pro-inflammatory cytokines and promotes CC development through inflammatory stimuli [63]. In CC cases, HOXA10 expression is reduced and associated with epithelial-mesenchymal transition (EMT). Its expression is positively correlated with E-cadherin and inversely correlated with vimentin expression [64]. Additionally, research by Ge showed that in CC patients with low HOXA10 expression, the abundance of Th2 cells was significantly increased, and PD-L1 expression was elevated, implicating HOXA10 in immune infiltration regulation [65]. These findings underscore the significant role of these transcription factors in regulating the progression and immune landscape of CC, particularly in the C0 fibroblast subtype.

In vitro studies have shown that SDC1 promotes the proliferation, migration, and invasion of CC cells. A reduction in SDC1 expression notably impaired the migration and invasion abilities of CC cells, suggesting that SDC1 could be a potential therapeutic target for CC. Multiplex immunohistochemistry and flow cytometry analyses revealed distinct spatial and phenotypic heterogeneity of fibroblasts across tumor, junctional, and normal zones, with cancer-associated fibroblasts exhibiting elevated expression of activation markers (α-SMA, Calponin, Vimentin, Fibronectin) predominantly in the tumor zone, while MYH11 expression was highest in normal fibroblasts and decreased toward the tumor zone. These findings highlight a spatially regulated fibroblast activation trajectory and dynamic stromal remodeling within the tumor microenvironment.

## Conclusion

Based on the single-cell characterization of fibroblast subtypes in CC and the validation of apCAF scores through deconvolution and spatial transcriptomics, we propose a potential link between the C0 MYH11 + Fibroblasts and CC progression. C0 MYH11 + Fibroblasts appear to be more sensitive to malignant and adverse prognostic events in CC, while other subtypes may exhibit greater suppression. Additionally, the differences in stemness and metabolic regulation between C0 MYH11 + Fibroblasts and other subtypes could provide valuable insights into preventing the excessive deterioration of these fibroblasts, potentially progressing into CAFs. Furthermore, the interaction between fibroblasts and tumor cells in CC may involve the MDK-SDC1 pathway, which represents a potential mechanism for blocking the critical link between C0 MYH11 + Fibroblasts, tumor cells, and the tumor microenvironment. This pathway offers meaningful insights for CC treatment. In addition, the transcriptional regulatory network identified in this study may be a good attempt to serve as a target to slow the progression of CC and prevent further deterioration. We validated the predictive potential of the model by constructing a CC prognostic model using a large number of RNA-seq data and key markers of fibroblast subtypes. The survival curve of SDC1 expression in tumor cells is particularly noteworthy. Finally, using in vitro experiments, we verified that the target protein SDC1 in tumor cells is influenced by C0 MYH11 + fibroblasts and may play an active role in inhibiting CC progression and immunosuppression.

In conclusion, our findings may provide new perspectives and potentially possible references for early prevention, immunotherapy, and suppression of CC progression and metastasis. By revealing the immunosuppressive microenvironment in CC, we increase the possibility of subsequent mining of potential immune targets and promising immunodrugs for further studies. Of course, further refinement and refinement of our study are needed to fully establish these findings.

## Supplementary Information

Supplementary Material 1: Fig. 1. CNV Levels Before and After Cell Type Annotation. (A) Before cell classification, tumor cells were selected from the epithelial cells based on the inferCNV results, showing CNV levels. (B) After cell type annotation, the CNV profiles of different fibroblast subtypes were displayed.

Supplementary Material 2: Fig. 2. Characteristic Landscapes and Deconvolution of Each Subtype's Naming Genes. (A) UMAP plots showed the expression differences of naming genes across the six fibroblast subtypes. (B) ST feature maps illustrated the spatial distribution of the six naming genes.(C-E) The deconvolution results of C0 MYH11 + fibroblasts were based on datasets GSE7803 and GSE52903 and in-house bulk RNA-seq data. (F) Different CNV scores corresponding to various CNV labels were presented.

Supplementary Material 3: Fig. 3. Associated CAF Scores in Fibroblast Subtypes. (A) A bubble plot displayed the differences in associated CAF scores across the six fibroblast subtypes. (B) UMAP plots illustrated the differences in five remaining associated CAF scores. (C) The density differences of the five associated CAF scores across the fibroblast subtypes were shown. (D) ST feature maps presented the spatial distribution of the five associated CAF scores. (E) Box plots compared the levels of the five associated CAF scores across different fibroblast subtypes.

Supplementary Material 4: Fig. 4. GO-BP Analysis of Differential Genes along Slingshot Trajectories and the Discovery and Validation of the Receptor Protein SDC1. (A) The differences in the expression of differentially expressed genes along the Lineage1 and Lineage2 trajectories were shown, with GO-BP analysis identifying distinct enrichment terms. (B) The differences in the strength of various signaling pathways through which C0 MYH11 + Fibroblasts interact with other cells were shown across different groups. (C) ST feature maps presented the spatial distribution of SDC1 as a receptor protein.

Supplementary Material 5: Fig. 5. Immune Infiltration Landscape in High and Low MFRS Groups. (A) A heatmap presented the immune cell infiltration profiles in the high MFRS and low MFRS groups. The colors indicated different cell types. (B) A boxplot showed the proportion of immune cell infiltration and the relative abundance of immune infiltrates in the high MFRS and low MFRS groups. (C) The correlation between immune cell types in different states was analyzed. (D) A bar chart illustrated the correlation between immune cell states and risk score genes across the high and low MFRS groups. (E) A heatmap displayed differences between the high and low MFRS groups in RiskScore, mRNA expression, estimate, cibersort, and xCell scores. (F) A boxplot showed the differences between the high and low MFRS groups in StroalScore, ImmuneScore, and ESTIMATEScore. (G) Tumor purity differences between the high and low MFRS groups were observed. (H) Differences in TIDE scores between the high and low MFRS groups were noted. (I) Tumor purity differences between the high and low age groups were found. (J) A waterfall plot displayed mutation frequencies in the high and low MFRS groups from the training cohort. The top row indicated mutation load for each sample, while the side column showed the overall percentage of mutated genes in each sample. (K) A bar chart displayed the CNV of 10 genes. Red represented chromosomal losses, blue represented chromosomal gains, and green represented no chromosomal alterations. (L) Differences in tumor mutation burden (TMB) between the high and low MFRS groups were observed. (M) A correlation between TMB and risk scores was found. (N–O) Kaplan–Meier survival curves were generated for high TMB versus low TMB groups, and for high-risk-high TMB, high-risk-low TMB, low-risk-high TMB, and low-risk-low TMB groups

Supplementary Material 6: Fig. 6. Kaplan–Meier Survival Curves for Key Genes. (A) Kaplan–Meier survival curves were generated for high and low expression groups of the following key genes: MYH11, KDM5B, CTNNB1, CD44, CUX1, TEAD1, HOXA10, PBX1, and FOSL2. The survival differences between the high and low expression groups were assessed for each gene.

Supplementary Material 7: Fig. 7. The proportion of all cell types in the ST feature map. (A) The pie chart in each spot shows the proportion of cells present at that location. (B) Differential expression of MYH11 in normal and tumor tissues in dataset GSE52903 and in-house bulk RNA-seq data.

Supplementary Material 8.Supplementary Table 1 The primer sequences

## Data Availability

The data for this study come from the ArrayExpress database (https://www.ebi.ac.uk/biostudies/arrayexpress). The accession number is E-MTAB-12305. The spatial transcriptome data from 10 × website (https://www.10xgenomics.com/). The bulk data were obtained from the TCGA website (https://portal.gdc.cancer.gov/) and UCSC Xena (https://xena.ucsc.edu/) and the GEO (https://www.ncbi.nlm.nih.gov/geo/). The GSE accession is GSE7803 and GSE52903. In-house bulk RNA-seq transcriptome data (3 tumor groups and 3 paracancerous control groups) were also included. All the data in this paper support the results of this study. Readers can contact the first author or corresponding author if they would like to further communicate relevant datasets and original sources.
