# Correction: Deciphering the tumor immune microenvironment: single-cell and spatial transcriptomic insights into cervical cancer fibroblasts

**DOI:** 10.1186/s13046-025-03597-z

**Published:** 2025-11-29

**Authors:** Zhiheng Lin, Youwei Zhou, Zhenran Liu, Wenyang Nie, Hengjie Cao, Shengnan Li, Xuanling Li, Lijun Zhu, Guangyao Lin, Yanyu Ding, Yi Jiang, Zuxi Gu, Lianwei Xu, Zhijie Zhao, Huabao Cai

**Affiliations:** 1 Department of Gynecology, Longhua Hospital, Shanghai University of Traditional Chinese Medicine, Shanghai, 200032 China; 2 Department of Obstetrics and Gynecology, the First Affiliated Hospital of Anhui Medical University, Hefei, 230032 China; 3 The First Clinical Medical College, Shandong University of Traditional Chinese Medicine, Jinan, 250014 China; 4 Department of Immunology, School of Basic Medical Sciences, Center for Big Dataand, Population Health of IHM, Anhui Medical University, Hefei, 230032 China; 5 Institute of Health and Medicine, Hefei Comprehensive National Science Center, Hefei Economic and Technological Development Zone, 4090 Susong Rd, HefeiHefei, Anhui 230601 China; 6 Experiment Center for Science and Technology, Shanghai University of Traditional Chinese Medicine, Shanghai, 201203 China; 7 Department of Laboratory Animal, School of Experimental Center of Science and Technology, Shanghai University of Traditional Chinese Medicine, Shanghai, 201203 China; 8 Department of Plastic and Reconstructive Surgery, School of Medicine, Shanghai Ninth People’ S Hospital, Shanghai Jiao Tong University, Shanghai, China; 9 Department of Neurosurgery, The First Affiliated Hospital of Anhui Medical University, 218 Jixi Rd, Hefei Shushan Zone, Hefei, Anhui 230032 China; 10 Center for Scientific Researchof, Anhui Medical University, Anhui Medical, Hefei, China

**Correction: J Exp Clin Cancer Res 44, 194 (2025)**

**https://doi.org/10.1186/s13046-025-03432-5**

Following publication of the original article [1], the authors identified error in the image of Fig. 12C where it was inadvertenty misused during figure preparation. The correct figure is given below:

**Incorrect Fig.** 12

**Fig. 12:**
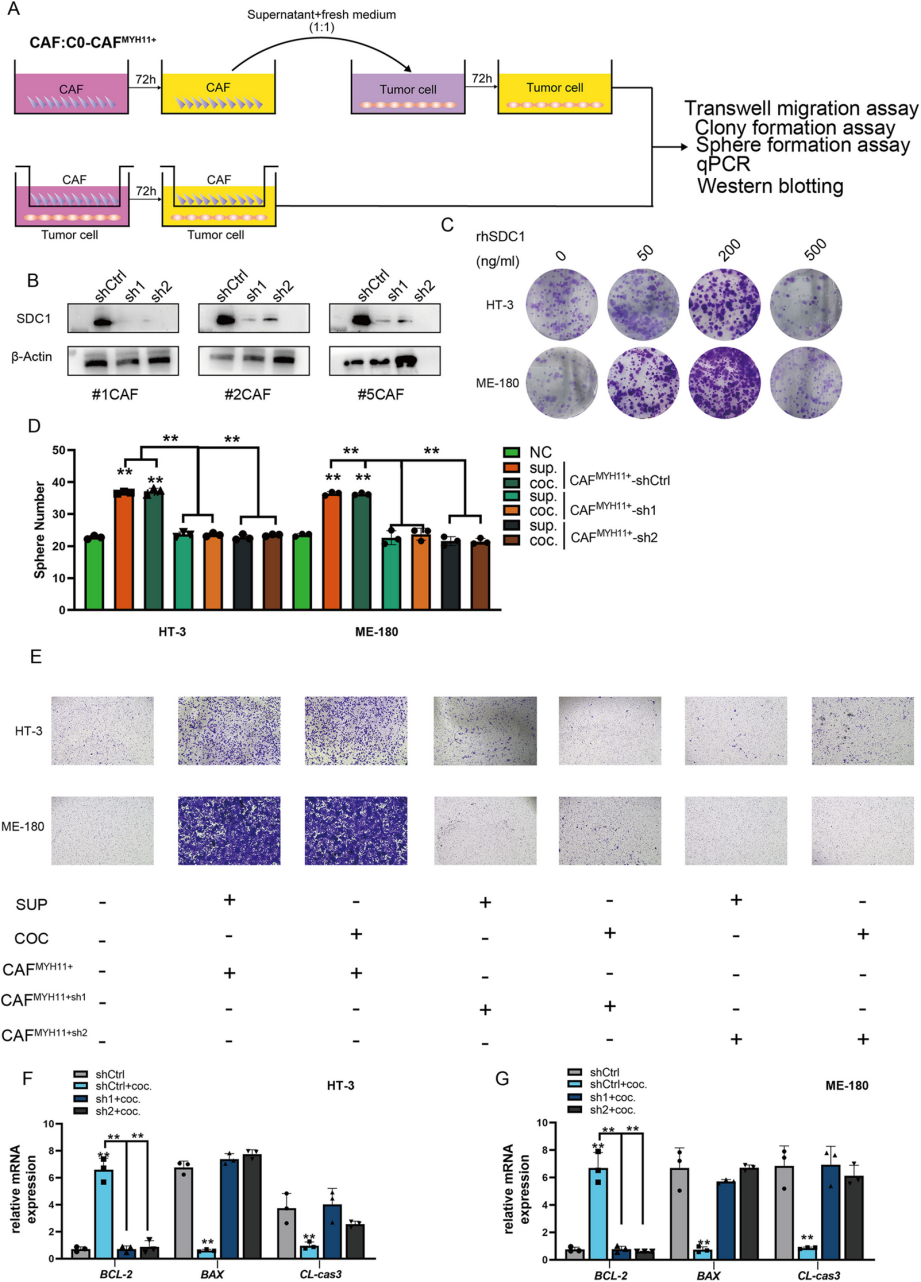
C0 MYH11 + CAF promotes tumor cell proliferation, migration, and inhibits apoptosis via soluble SDC1. **A** Experimental procedure for co-culture of C0 MYH11 + CAF with high- and low-invasive cervical cancer cells. Supernatants were collected and used to treat tumor cells. **B** shRNA-mediated silencing of SDC1 in C0 MYH11 + CAF resulted in reduced SDC1 expression. Western blot showing SDC1 levels in control and silenced groups. **C** Recombinant SDC1 treatment (200 ng/mL) promoted tumor cell proliferation, whereas 500 ng/mL inhibited proliferation. **D** Spheroid formation assay showing enhanced stemness and malignancy in tumor cells co-cultured with C0 MYH11 + CAF or treated with its supernatant. **E** Migration assay indicating increased migratory ability of tumor cells exposed to C0 MYH11 + CAF or its culture supernatant. **F-G** qRT-PCR analysis of apoptosis-related gene expression showing upregulation of anti-apoptotic and downregulation of pro-apoptotic genes in tumor cells treated with C0 MYH11 + CAF supernatant, suggesting reduced apoptosis

**Correct Fig.** 12

**Fig. 12:**
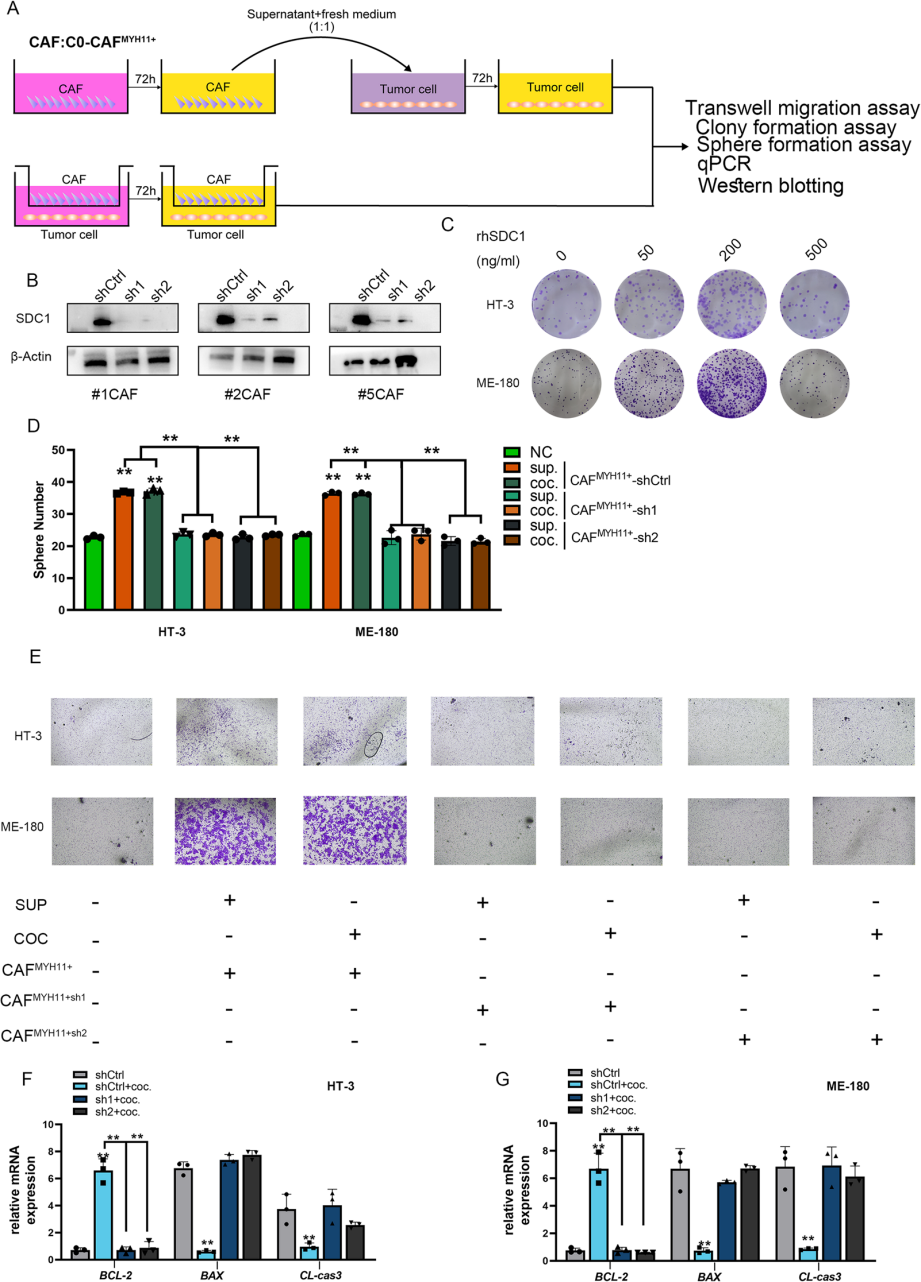
C0 MYH11 + CAF promotes tumor cell proliferation, migration, and inhibits apoptosis via soluble SDC1. **A** Experimental procedure for co-culture of C0 MYH11 + CAF with high- and low-invasive cervical cancer cells. Supernatants were collected and used to treat tumor cells. **B** shRNA-mediated silencing of SDC1 in C0 MYH11 + CAF resulted in reduced SDC1 expression. Western blot showing SDC1 levels in control and silenced groups. **C** Recombinant SDC1 treatment (200 ng/mL) promoted tumor cell proliferation, whereas 500 ng/mL inhibited proliferation. **D** Spheroid formation assay showing enhanced stemness and malignancy in tumor cells co-cultured with C0 MYH11 + CAF or treated with its supernatant. **E** Migration assay indicating increased migratory ability of tumor cells exposed to C0 MYH11 + CAF or its culture supernatant. **F-G** qRT-PCR analysis of apoptosis-related gene expression showing upregulation of anti-apoptotic and downregulation of pro-apoptotic genes in tumor cells treated with C0 MYH11 + CAF supernatant, suggesting reduced apoptosis

The correction dos not compromise the validity of the conclusions and the overall content of the article. The author group has been updated above and the original article [1] has been corrected.
