# Supplementary figures and images for: Deciphering the tumor immune microenvironment: single-cell and spatial transcriptomic insights into cervical cancer fibroblasts
Source: J Exp Clin Cancer Res. 2025 Jul 5;44:194. doi: 10.1186/s13046-025-03432-5 (PMC12228347; doi:10.1186/s13046-025-03432-5)

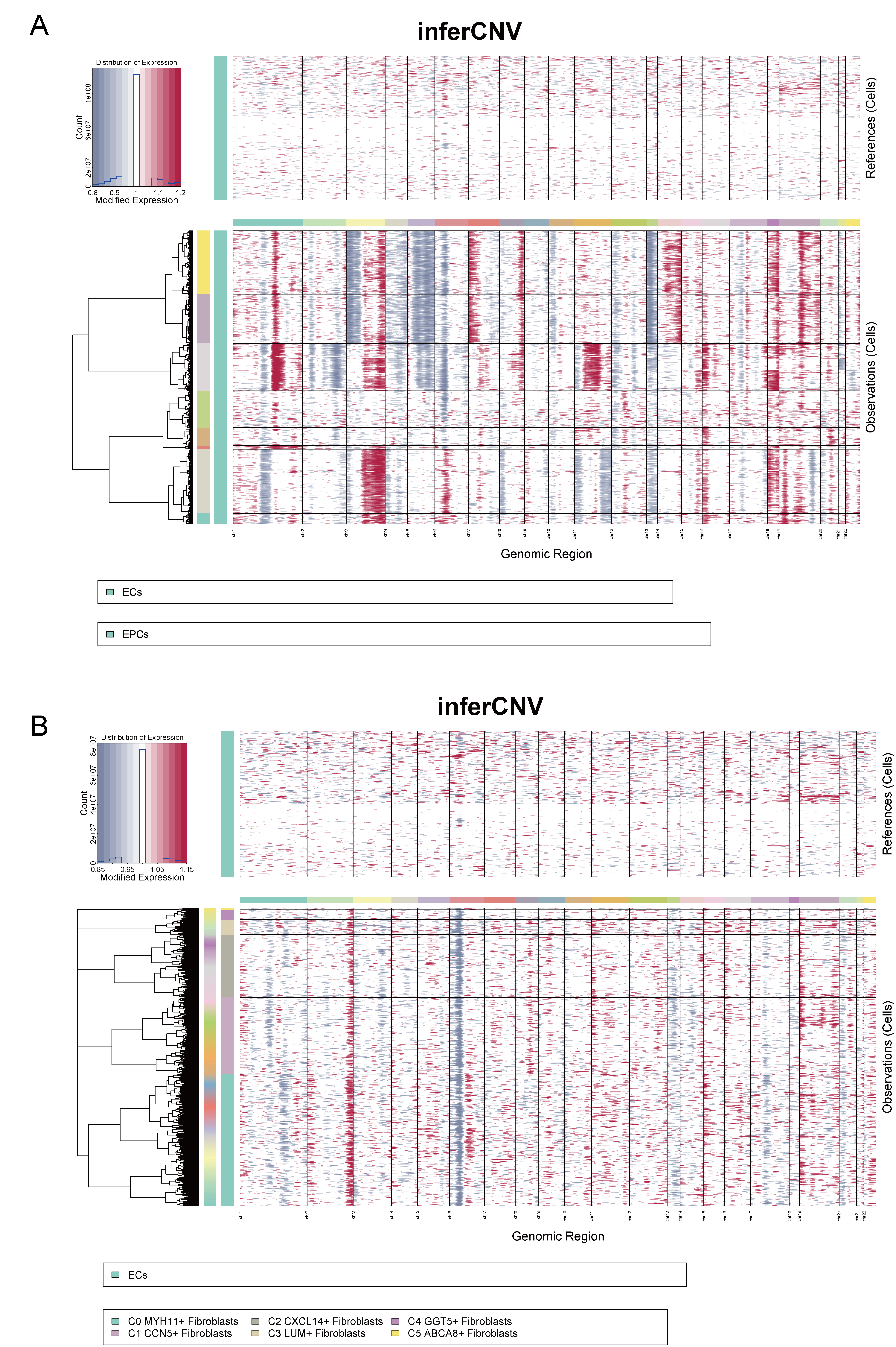

Supplement: Supplementary file 1 — Supplementary Material 1: Fig. 1. CNV Levels Before and After Cell Type Annotation. (A) Before cell classification, tumor cells were selected from the epithelial cells based on the inferCNV results, showing CNV levels. (B) After cell type annotation, the CNV profiles of different fibroblast subtypes were displayed. [file 13046_2025_3432_MOESM1_ESM.tif]

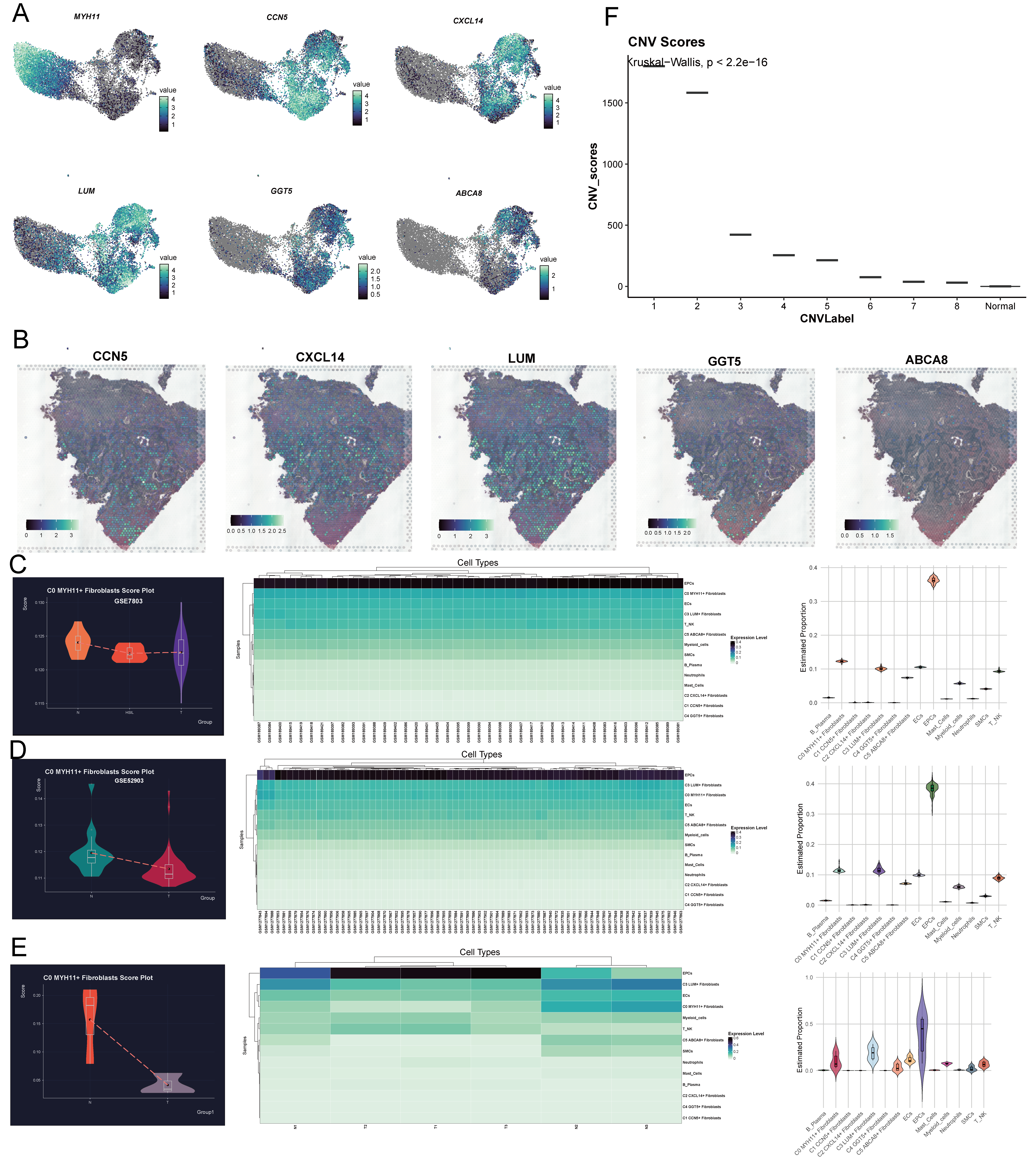

Supplement: Supplementary file 2 — Supplementary Material 2: Fig. 2. Characteristic Landscapes and Deconvolution of Each Subtype's Naming Genes. (A) UMAP plots showed the expression differences of naming genes across the six fibroblast subtypes. (B) ST feature maps illustrated the spatial distribution of the six naming genes.(C-E) The deconvolution results of C0 MYH11 + fibroblasts were based on datasets GSE7803 and GSE52903 and in-house bulk RNA-seq data. (F) Different CNV scores corresponding to various CNV labels were presented. [file 13046_2025_3432_MOESM2_ESM.tif]

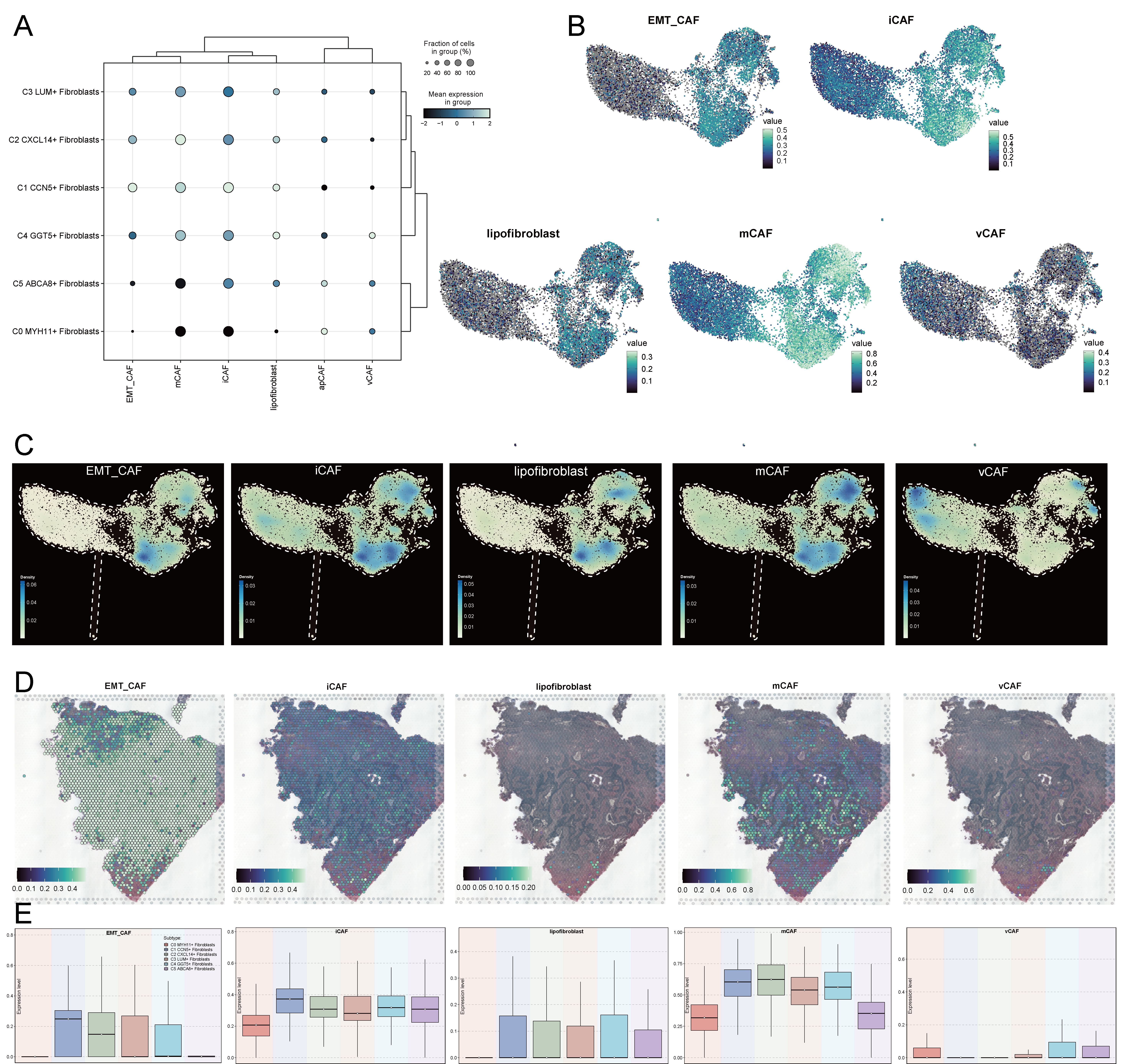

Supplement: Supplementary file 3 — Supplementary Material 3: Fig. 3. Associated CAF Scores in Fibroblast Subtypes. (A) A bubble plot displayed the differences in associated CAF scores across the six fibroblast subtypes. (B) UMAP plots illustrated the differences in five remaining associated CAF scores. (C) The density differences of the five associated CAF scores across the fibroblast subtypes were shown. (D) ST feature maps presented the spatial distribution of the five associated CAF scores. (E) Box plots compared the levels of the five associated CAF scores across different fibroblast subtypes. [file 13046_2025_3432_MOESM3_ESM.tif]

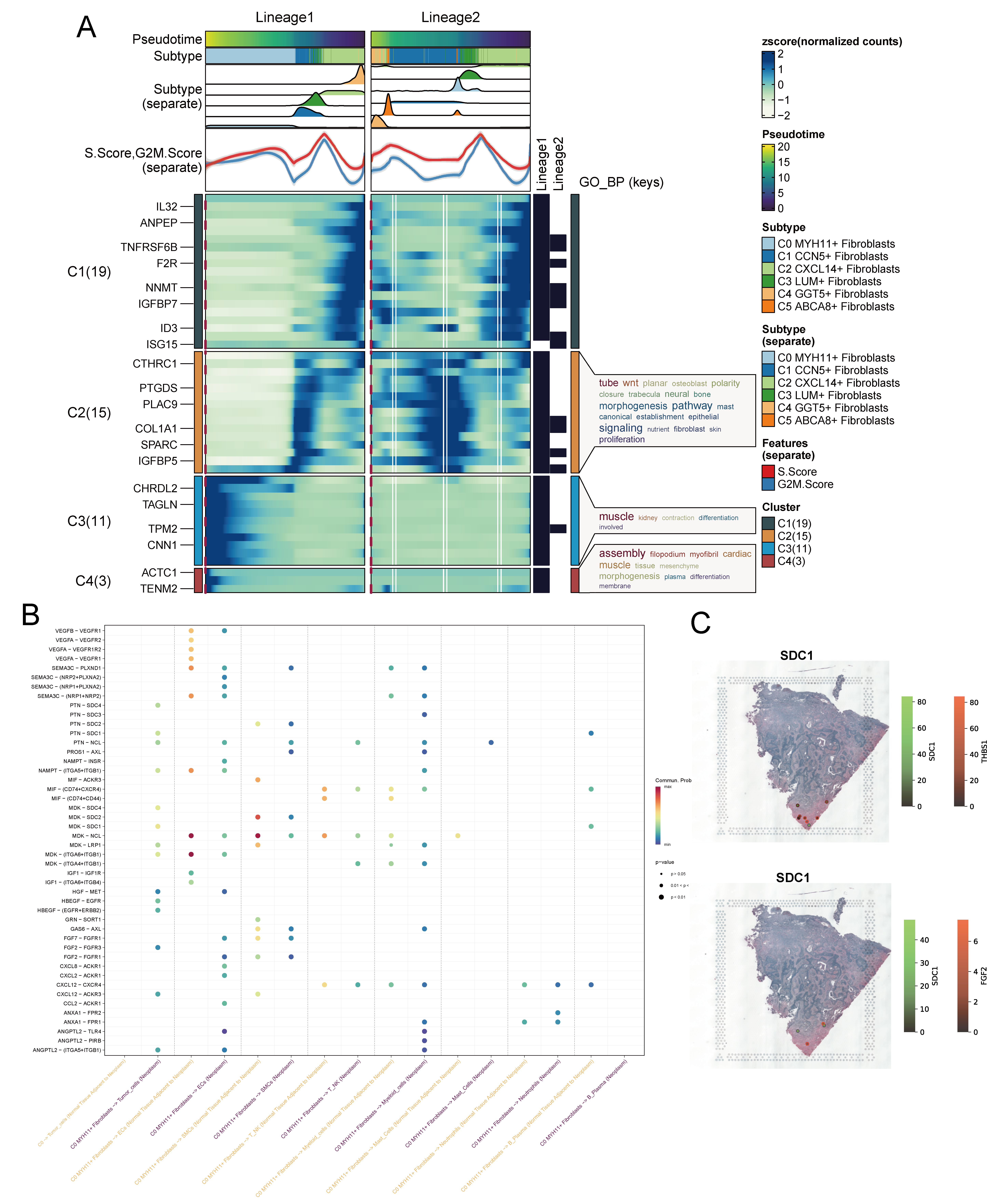

Supplement: Supplementary file 4 — Supplementary Material 4: Fig. 4. GO-BP Analysis of Differential Genes along Slingshot Trajectories and the Discovery and Validation of the Receptor Protein SDC1. (A) The differences in the expression of differentially expressed genes along the Lineage1 and Lineage2 trajectories were shown, with GO-BP analysis identifying distinct enrichment terms. (B) The differences in the strength of various signaling pathways through which C0 MYH11 + Fibroblasts interact with other cells were shown across different groups. (C) ST feature maps presented the spatial distribution of SDC1 as a receptor protein. [file 13046_2025_3432_MOESM4_ESM.tif]

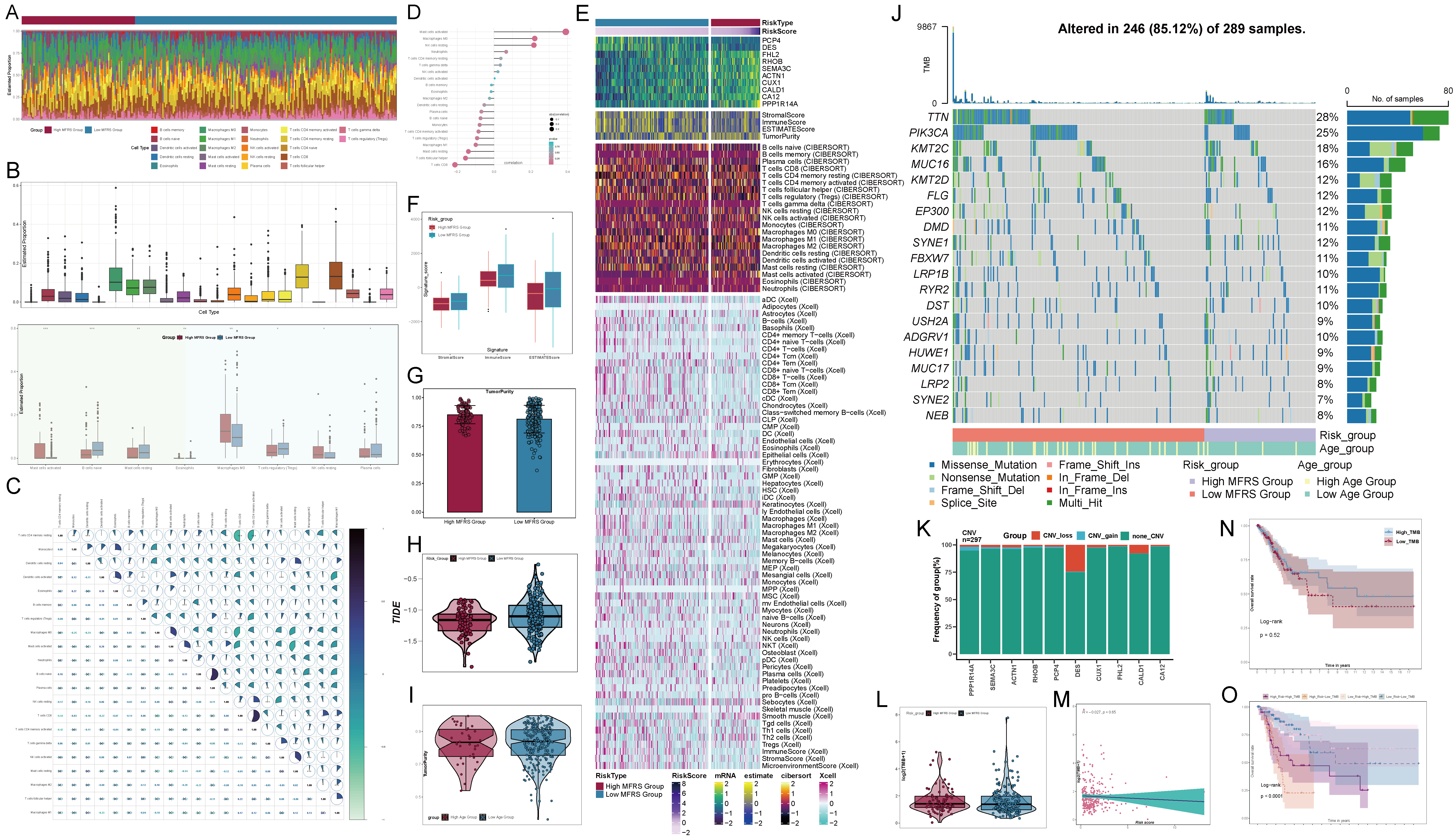

Supplement: Supplementary file 5 — Supplementary Material 5: Fig. 5. Immune Infiltration Landscape in High and Low MFRS Groups. (A) A heatmap presented the immune cell infiltration profiles in the high MFRS and low MFRS groups. The colors indicated different cell types. (B) A boxplot showed the proportion of immune cell infiltration and the relative abundance of immune infiltrates in the high MFRS and low MFRS groups. (C) The correlation between immune cell types in different states was analyzed. (D) A bar chart illustrated the correlation between immune cell states and risk score genes across the high and low MFRS groups. (E) A heatmap displayed differences between the high and low MFRS groups in RiskScore, mRNA expression, estimate, cibersort, and xCell scores. (F) A boxplot showed the differences between the high and low MFRS groups in StroalScore, ImmuneScore, and ESTIMATEScore. (G) Tumor purity differences between the high and low MFRS groups were observed. (H) Differences in TIDE scores between the high and low MFRS groups were noted. (I) Tumor purity differences between the high and low age groups were found. (J) A waterfall plot displayed mutation frequencies in the high and low MFRS groups from the training cohort. The top row indicated mutation load for each sample, while the side column showed the overall percentage of mutated genes in each sample. (K) A bar chart displayed the CNV of 10 genes. Red represented chromosomal losses, blue represented chromosomal gains, and green represented no chromosomal alterations. (L) Differences in tumor mutation burden (TMB) between the high and low MFRS groups were observed. (M) A correlation between TMB and risk scores was found. (N–O) Kaplan–Meier survival curves were generated for high TMB versus low TMB groups, and for high-risk-high TMB, high-risk-low TMB, low-risk-high TMB, and low-risk-low TMB groups [file 13046_2025_3432_MOESM5_ESM.tif]

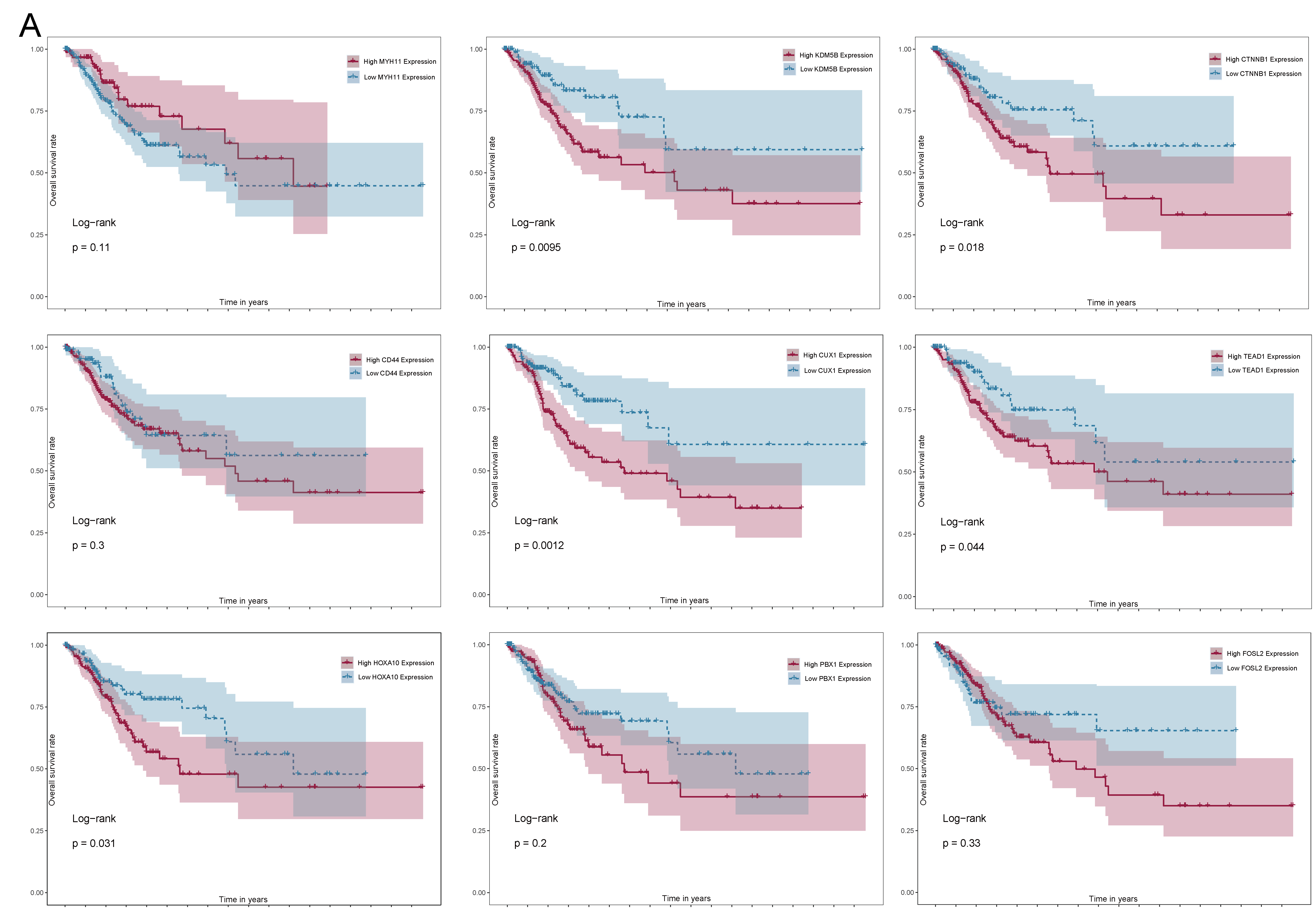

Supplement: Supplementary file 6 — Supplementary Material 6: Fig. 6. Kaplan–Meier Survival Curves for Key Genes. (A) Kaplan–Meier survival curves were generated for high and low expression groups of the following key genes: MYH11, KDM5B, CTNNB1, CD44, CUX1, TEAD1, HOXA10, PBX1, and FOSL2. The survival differences between the high and low expression groups were assessed for each gene. [file 13046_2025_3432_MOESM6_ESM.tif]

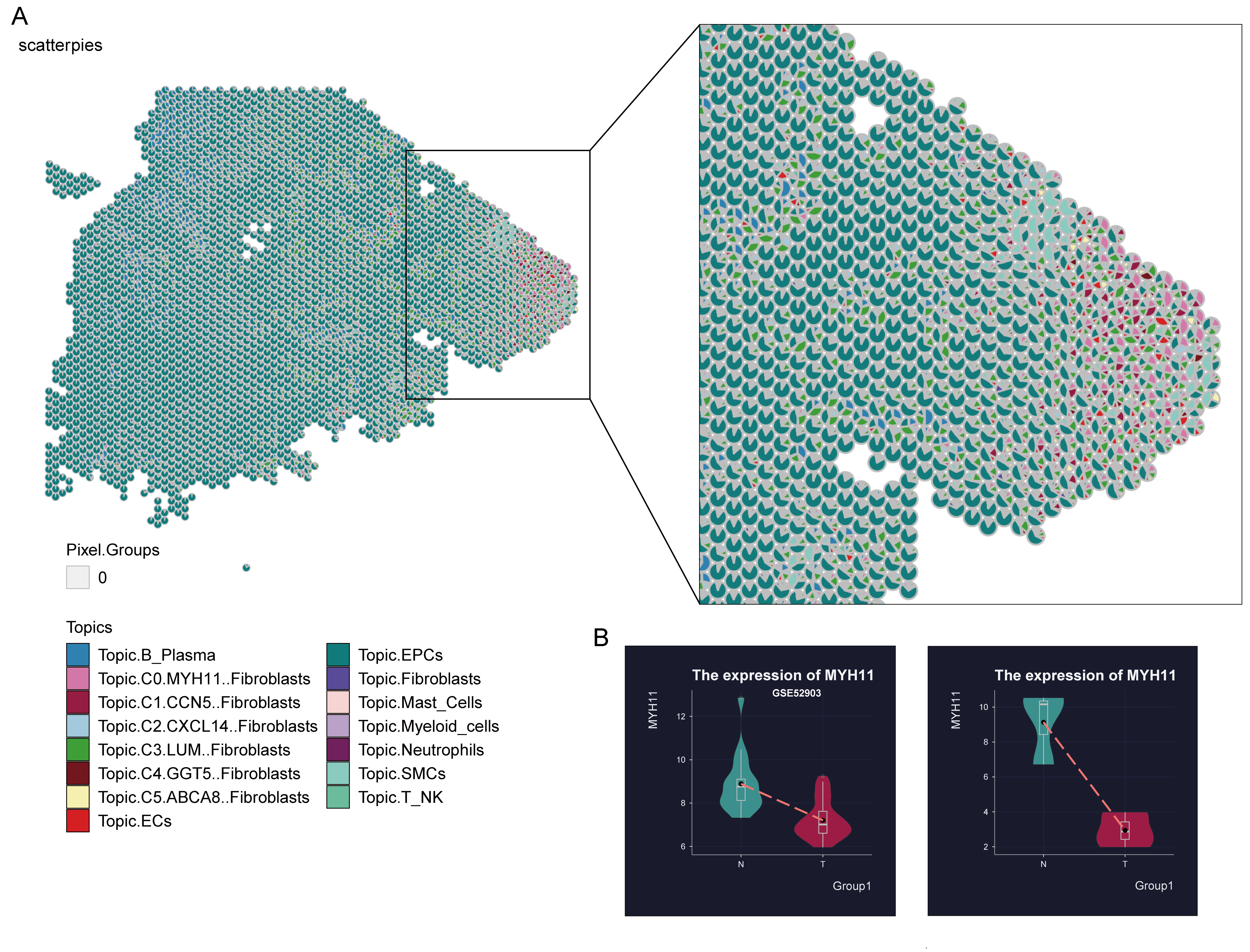

Supplement: Supplementary file 7 — Supplementary Material 7: Fig. 7. The proportion of all cell types in the ST feature map. (A) The pie chart in each spot shows the proportion of cells present at that location. (B) Differential expression of MYH11 in normal and tumor tissues in dataset GSE52903 and in-house bulk RNA-seq data. [file 13046_2025_3432_MOESM7_ESM.tif]
