# Supplementary material for: Deciphering the tumor immune microenvironment: single-cell and spatial transcriptomic insights into cervical cancer fibroblasts
Source: J Exp Clin Cancer Res. 2025 Jul 5;44:194. doi: 10.1186/s13046-025-03432-5 (PMC12228347; doi:10.1186/s13046-025-03432-5)
Supplement: Supplementary file 8 — Supplementary Material 8.Supplementary Table 1 The primer sequences [file 13046_2025_3432_MOESM8_ESM.docx]

**Supplementary Table 1 The primer sequences**

| **Gene Name** | **Direction** | **Primer Sequence (5' → 3')** | **Amplicon Length (bp)** | **Notes** |
| --- | --- | --- | --- | --- |
| **SDC1** | Forward | CCTGGGCTACCTGTTCATCC | ~120 | Human reference |
|  | Reverse | GGTGTTGGTGCTGTTGTAGG |  |  |
| **BCL-2** | Forward | GGTGGGGTCATGTGTGTGG | ~100 | Human reference |
|  | Reverse | CGGTTCAGGTACTCAGTCATCC |  |  |
| **BAX** | Forward | CCCGAGAGGTCTTTTTCCGAG | ~150 | Human reference |
|  | Reverse | CCAGCCCATGATGGTTCTGAT |  |  |
| **Cleaved-Caspase 3** | Forward | TGTCATCTCGCTCTGGTACG | ~120 | Human reference |
|  | Reverse | AAATGACCCCTTCATCACCAAC |  |  |
